# Longitudinal analysis of neutralizing antibodies against SARS-CoV-1 and different SARS-CoV-2 strains in breakthrough and unvaccinated COVID-19 patients in Thailand (2021-2022)

**DOI:** 10.1038/s41598-025-33388-7

**Published:** 2026-01-03

**Authors:** Prapassorn Poolchanuan, Vichapon Tiacharoen, Adul Dulsuk, Rungnapa Phunpang, Chakkaphan Runcharoen, Thitiya Boonprakob, Onura Hemtong, Suchada Chowplijit, Vachara Chuapaknam, Tanaya Siripoon, Watcharapong Piyaphanee, Le Van Tan, Susanna Dunachie, Chee Wah Tan, Lin-Fa Wang, Wasun Chantratita, Viravarn Luvira, Narisara Chantratita, SEACOVARIANTS, Nguyen To Anh, Nguyen Thi Thu Hong, Truong Hoang Chau Truc, Nguyen Thi Han Ny, Do Duong Kim Han, Le Kim Thanh, Lam Anh Nguyet, Cao Thu Thuy, Le Nguyen Truc Nhu, Tran Tan Thanh, Nguyen To Anh, Lam Minh Yen, Vu Thi Ty Hang, Pham Tieu Kieu, Vo Tan Hoang, Nguyen Thi Thao, Mary Chambers, Vu Duy Thanh, Tran Chieu Hoang, CLouise Thwaites, Guy Thwaites, H.Rogier van Doorn, Trinh Son Tung, Raph L Hamers, Anuraj Shankar, Juthathip Mongkolsapaya, Gavin Screaton, Aiete Dijokaite-Guraliuc, Raksha Das, Chang Liu, Piyada Supasa, Muneeswaran Selvaraj, Paul Klenerman, EYvonne Jones, David I Stuart, Barbara Kronsteiner-Dobramysl, Martha Zewdie, Priyanka Abraham, Jennifer Hill, Wee Chee Yap, Beng Lee Lim, Suwarti Suwarti, Yanie Tayipto, Eva Simarmata, Ragil Dien, Wanwisa Dejnirattisai, Warangkana Chantima, Apirak Junpirom, Sopon Iamsirithaworn, Nicholas P.J. Day, Phaik Yeong Cheah, Tassawan Poomchaichote, Kanpong Boonthaworn, Nghiem My Ngoc, Alba Grifoni, Alessandro Sette

**Affiliations:** 1 Department of Microbiology and Immunology, Faculty of Tropical Medicine, Mahidol University, 420/6 Rajvithi Road, Bangkok, 10400 Thailand; 2 Center for Medical Genomics, Faculty of Medicine Ramathibodi Hospital, Mahidol University, Bangkok, Thailand; 3 Faculty of Medical Technology, Huachiew Chalermprakiet University, Samut Prakan, Thailand; 4 Prachatipat Hospital, Pathum Thani, Thailand; 5 Vichaivej International Hospital, Samut Sakhon, Thailand; 6 Department of Clinical Tropical Medicine, Faculty of Tropical Medicine, Mahidol University, Bangkok, Thailand; 7 Vaccine Trial Centre, Faculty of Tropical Medicine, Mahidol University, Bangkok, Thailand; 8 Faculty of Tropical Medicine, Thai Travel Clinic, Hospital for Tropical Diseases, Mahidol University, Bangkok, Thailand; 9 Oxford University Clinical Research Unit, Ho Chi Minh City, Vietnam; 10 Centre for Tropical Medicine and Global Health University of Oxford, Oxford, United Kingdom; 11 NDM Centre for Global Health Research, Nuffield Department of Clinical Medicine, University of Oxford, Oxford, United Kingdom; 12 Faculty of Tropical Medicine, Mahidol-Oxford Tropical Medicine Research Unit, Mahidol University, Bangkok, Thailand; 13 Programme in Emerging Infectious Diseases, Duke-NUS Medical School, Singapore, Singapore; 14 Department of Microbiology and Immunology, Infectious Diseases Translational Research Programme, Yong Loo Lin School of Medicine, National University of Singapore, Singapore, Singapore; 15 Oxford University Clinical Research Unit, Ha Noi, Vietnam; 16 University of Oxford, Oxford, United Kingdom; 17 Oxford University Clinical Research Unit, Jakarta, Indonesia; 18 Faculty of Medicine, Siriraj Hospital, Mahidol University, Bangkok, Thailand; 19 Ministry of Public Health, Nonthaburi, Thailand; 20 Hospital for Tropical Diseases, Ho Chi Minh City, Vietnam; 21 La Jolla Institute for Immunology, San Diego, California United States

**Keywords:** Diseases, Immunology, Microbiology

## Abstract

The emergence of SARS-CoV-2 variants that evade immune responses poses challenges to effective prevention. We prospectively enrolled 111 COVID-19 patients in Thailand (2021-2022), who received homologous or heterologous vaccines or were unvaccinated. Plasma neutralizing antibody (nAb) levels against SARS-CoV-1 and 13 SARS-CoV-2 strains were measured using a multiplex surrogate virus neutralization test (sVNT) assay within a year. nAb levels increased in two weeks, showing strong inhibition against ancestral SARS-CoV-2 and non-omicron variants, but not against SAR-CoV-1 and lower responses to omicron variants. nAb levels declined by the day 60. Breakthrough patients with heterologous vaccines had higher nAb levels compared to other groups. nAb levels were lower in breakthrough patients with pneumonia than those with other conditions. Notably, breakthrough patients aged ≥60 showed rapid declines in antibody levels. Our findings highlight diverse immune responses influenced by immunization, age, and clinical conditions, underscoring the need for tailored vaccination strategies against evolving variants.

**Supplementary Information:**

The online version contains supplementary material available at 10.1038/s41598-025-33388-7.

## Introduction

SARS-CoV-1 and SARS-CoV-2 are both members of the genus *Betacoronavirus*, and the former was responsible for the outbreak of severe acute respiratory syndrome (SARS) in 2002–2003, primarily in China. SARS-CoV-2 is the causative agent of the ongoing Coronavirus Disease 2019 (COVID-19) pandemic, first identified in Wuhan, China, in late 2019^1^. Both viruses have single-stranded RNA genomes that share approximately 79% genetic similarity. The spike (S) protein has been a primary focus of research due to its role in viral entry into host cells^2^. SARS-CoV-1 and SARS-CoV-2 use the ACE2 receptor for cell entry via their spike protein’s receptor-binding domain (RBD), which differs in their respective amino acid sequences and structural features^3^. The emerging SARS-CoV-2 variants have raised particular attention due to their increased transmissibility, disease severity, and ability to evade immunity conferred by previous infection or vaccination^4^. In 2023, the omicron XBB.1.5 and XBB.1.16 variants were the dominant circulating variants in Thailand and Asia^5–7^. These dominant variants have replaced previously outbreak strains (such as ancestral, alpha, beta, gamma, delta, lambda, Mu and delta plus strains) and circulated worldwide, including in the American, European, and African continents^8–11^. SARS-CoV-2 undergoes continued mutations, leading to the emergence of numerous subvariants.

Antibodies, whether from vaccination or natural infection, protect against SARS-CoV-2 through their pivotal roles in the disease prevention and viral clearance processes, as well as in reducing disease severity and lowering the risk of hospitalization^12,13^. Newly emerging SARS-CoV-2 variants undergo mutations that enable them to evade the immune response, thus compromising immunity against these emerging variants of concern (VOCs)^14,15^. The first vaccine administered to the people in Thailand and other Southeast Asian countries was the CoronaVac vaccine. Subsequently, various other types of vaccines such as the ChAdOx1-S vaccine, BNT162b2 or mRNA-1273 vaccine, and protein subunit vaccine, have been administered to the Southeast Asia population to prevent infection^16–19^. Thus, most Asian populations have received various mixed-vaccine regimens that were provided by their respective governments. Meanwhile, populations in European and American countries have predominantly been immunized using ChAdOx1-S, BNT162b2, and mRNA-1273 vaccines^20,21^. Immune evasion against VOCs was observed in convalescent patients, in whom the antibody levels against BA.1, BA.2, BA.4/5, XBB.1.5, CH.1.1, and CA.3.1 variants decreased by 3-, 1.8-, 3-, 9.6-, 12.6-, and 13.3-fold, respectively, compared to the ancestral strain^14–16^. Variant XBB.1.16 was one of the dominant variants in Thailand and many other countries in 2023, and it is characterized by two substitutions in the S protein: E180V and T478R. The concentration of antibodies against this variant has been reported to be approximately 18 times lower than that observed for the alpha variant^6^.

Previous research has demonstrated that levels of both neutralizing anibody (nAb) and binding antibody against the omicron BA.1 variant are lower than those against the ancestral strain among vaccinated individuals^22^. Antibody responses to SARS-CoV-2 have been established to be correlated to various factors such as underlying health conditions, age, and disease severity^23–25^. However, only a few longitudinal investigations into the immune response against coronaviruses and newly emerging VOCs within the Asian demographic have been conducted^26–29^. Thus, there is a significant gap in understanding the antibody response and its effectiveness in conferring protection against these variants, which raises concerns regarding the ongoing evolution and potential resurgence of SARS-CoV-2. Research on the efficacy of vaccine regimens in mitigating the impact of SARS-CoV-2 variants in Thailand has been limited. Moreover, the data on the immune response against VOCs among individuals with diverse health conditions are insufficient. In addition, novel high-throughput neutralization assays have not been widely used, thereby limiting our ability to assess immune responses effectively.

This study is an integral component of the initiative aimed at investigating the immune response to SARS-CoV-2 variants within Southeast Asian populations (SEACOVARIANTS) (https://wellcomeopenresearch.org/articles/9-181). In this collaborative endeavor involving partners from Thailand, Vietnam, Indonesia, Singapore, the UK, and the US, we prospectively enrolled 111 COVID-19 patients to assess the nAb response against SARS-CoV-1, SARS-CoV-2 ancestral strain, and 12 SARS-CoV-2 variants. Plasma samples were systematically collected from Thai patients at six time points over 1 year (days 0, 14, 28, 60, 180, and 365). The nAb levels were measured using a novel high-throughput surrogate virus neutralization test (multiplex sVNT), which was previously shown to have a high correlation with the gold-standard live virus neutralization assay^30–34^. In our analysis, we compared the nAb levels in patients who received different types of vaccines and those who were unvaccinated.

## Results

### Demographics and characteristics of COVID-19 patients

**Fig. 1:**
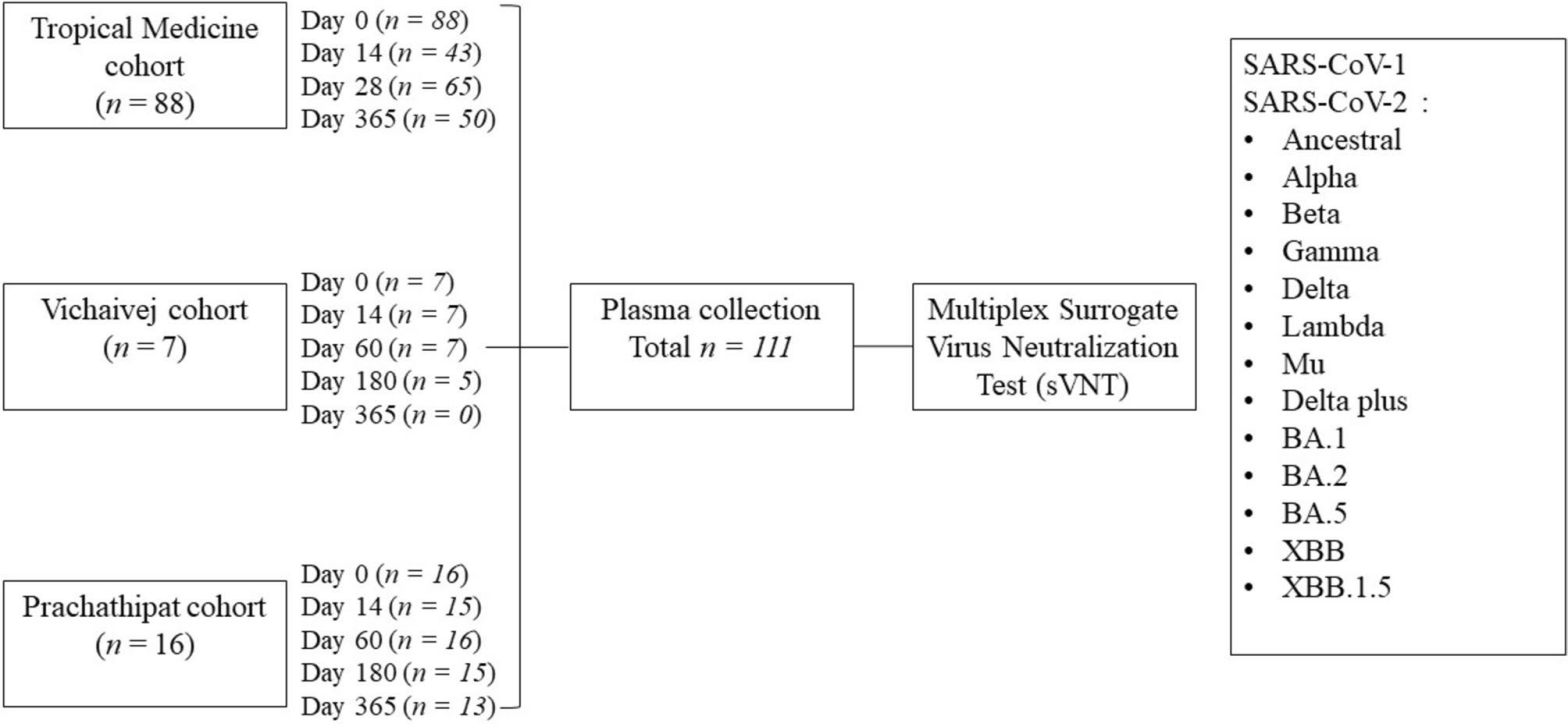
A study flow chart depicting the prospective enrollment of 111 COVID-19 patients from three hospitals in central Thailand spanning from July 2021 to December 2022. Plasma samples were collected from enrolled patients, and their neutralizing antibody (nAb) levels against SARS-CoV-1, the SARS-CoV-2 ancestral strain, and 12 SARS-CoV-2 variants were assessed using the multiplex surrogate virus neutralization test (sVNT).

One hundred and eleven COVID-19 patients were recruited from three hospitals in Thailand from July 2021 to December 2022 (Figure 1). This study period covered the delta and omicron infection waves in Thailand, spanning from the middle of 2021 until the end of 2022. In addition, the vaccines administered during the study period were ancestral strain-based vaccines. The demographics and characteristics of the patients are shown in Table 1. The patients’ median age was 53 years (interquartile range [IQR], 41–63 years), 45/111 (40.5%) were male, and 17/111 (15.3%) were not vaccinated against COVID-19 prior to SARS-CoV-2 infection. Ninety-four patients (84.7%) received 1–5 doses (median = 2 doses, IQR = 2–4 doses) of the inactivated virus vaccine (CoronaVac or BBIBP-CorV), viral vector vaccine (ChAdOx1-S), or mRNA vaccine (BNT162b2 or mRNA-1273), and among them, 10/94 (10.6%) had received a homologous inactivated virus vaccine (CoronaVac or BBIBP-CorV), 31/94 (33.0%) had received a homologous viral vector vaccine (ChAdOx1-S), 4/94 (4.3%) had received a homologous mRNA vaccine (BNT162b2 or mRNA-1273), and 49/94 (52.1%) had received a heterologous vaccine. The type and combination of vaccine regimens the patients received are shown in Supplementary Table S1. Sixty-four patients (57.7%) had underlying conditions. Of the 111 patients, 35 (31.5%) had hypertension, 30 (27.0%) had dyslipidemia, 18 (16.2%) had diabetes mellitus, and 20 (18%) had obesity (BMI ≥ 30). The median (IQR) length of hospital stays for unvaccinated patients (10 [9–15] days) was longer than that for the breakthrough patients (8 [6–11] days) (*p* = 0.006). Of the patients, 41 (36.9%) had pneumonia while 64 (57.7%) experienced lung involvement and complications related to COVID-19 infection. None of the patients died in the admission.

**Table 1.** Demographics and clinical characteristics of COVID-19 patients

**Characteristics**	**COVID-19 patients**^**a**^ **(N = 111)**
**Demographics**	
Age in years, median (IQR)	53 (41–63)
Male, number, %	45 (40.5)
Female, number, %	66 (59.5)
**Vaccination history, number, %**	
Unvaccinated	17 (15.3)
Homologous inactivated vaccine (1–2 doses)	10 (9.0)
Homologous viral vector vaccine (1–2 doses)	31 (27.9)
Homologous mRNA vaccine (2–3 doses)	4 (3.6)
Heterologous vaccine (2–5 doses) *	49 (44.1)
**Pre-existing conditions, number, %**	
No underlying disease	47 (42.3)
Hypertension	35 (31.5)
Dyslipidemia	30 (27.0)
Diabetes mellitus	18 (16.2)
Chronic heart disease	8 (7.2)
BMI ≥ 30	20 (18.0)
Chronic hematologic disease	3 (2.7)
Asthma	4 (3.6)
Cancer	1 (0.9)
Rheumatologic disease	5 (4.5)
**Day to enrollment, median (IQR)** **Duration of hospital stay, day, median (IQR)**	1 (0–2)
Unvaccinated patients Vaccinated patients	10 (9–15) 8 (6–11)
**Pneumonia**	41 (36.9)
**Organ involved and complications, N (%)**	
Lung	64 (57.7)
Liver	13 (11.7)
Kidney	5 (4.5)
**Death**	0 (0)

^*^Heterologous vaccine regimens consist of combinations of 2–5 doses of inactivated, viral vector, and mRNA vaccines (see supplementary Table S1)

^a^ Neutralizing antibody data for 111 COVID-19 patients were partially obtained from a previously published dataset^33^.

### Neutralizing antibody (nAb) levels against SARS-CoV-1 and SARS-CoV-2 strains in COVID-19 patients

**Fig. 2:**
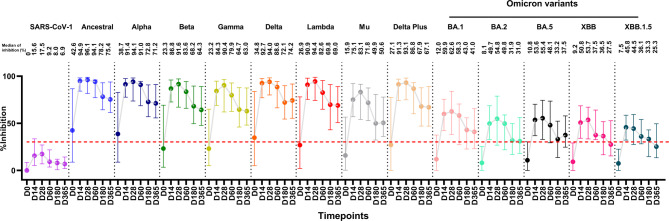
Neutralizing antibodies (nAbs) against SARS-CoV-1, SARS-CoV-2 ancestral strain and its variants over 1 year in COVID-19 patients. The nAb were determined using the multiplex surrogate virus neutralization test (sVNT) assay on plasma samples from 111 COVID-19 patients on days 0 (N = 111), 14 (N = 65), 28 (N = 65), 60 (N = 23), 180 (N = 20), and 365 (N = 63) after enrollment. A mixed-effects model was used to compare the median differences between groups. Numbers on the graph represent the median percent inhibition of nAbs. A percentage of inhibition values of less than 30% (indicated by the dotted red line) were considered negative results. ***, *p <* 0.001; **, p < 0.010, and *, *p <* 0.050. The bars in the graphs represent the interquartile range (IQR). Neutralizing antibody data for 111 COVID-19 patients were partially obtained from a previously published dataset^33^.

Multiplex sVNT was used to determine levels of plasma nAbs in all COVID-19 patients at different time points within 1 year. The levels of nAbs (expressed as % inhibition) for 14 RBDs of SARS-CoV-1, SARS-CoV-2 ancestral strain, and twelve SARS-CoV-2 variants were compared. The variants included seven non-omicron variants (alpha, beta, gamma, delta, lambda, mu, delta plus) and five omicron variants (BA.1, BA.2, BA.5, XBB, and XBB.1.5) **(**Figure 2, Supplementary Table S2**)**. Statistical comparisons of nAb levels against SARS-CoV-1 and SARS-CoV-2 variants at each time point, using mixed-effect model, are presented in Supplementary Table S3. Because of the home isolation policy and relocation of some patients, we were unable to follow-up some of the COVID-19 patients at certain time points.

The median levels of nAbs against SARS-CoV-1 in COVID-19 patients for all time points were below the cut-off point of 30% inhibition. For nAbs against the 13 SARS-CoV-2 strains, the median levels of inhibition at day 0 were low at 42.6%, 38.7%, and 34.8% for ancestral, alpha and delta, respectively, and below 30% for beta, gamma, lambda, Mu, delta plus, and all omicron variants (BA.1, BA.2, BA.5, XBB and XBB.1.5). On day 14, the median levels of inhibition against all SARS-CoV-2 strains increased significantly (*p* < 0.001, for all comparisons between day 0 and day 14). However, the median inhibition on day 14 against the omicron variants (BA.1, BA.2, BA.5, XBB, and XBB.1.5 variants) was significantly lower compared to that of the non-omicron variants (ancestral, alpha, beta, gamma, delta, lambda, Mu, delta plus) (50.8% vs. 91.1%, *p* < 0.001). The levels of nAbs against all SARS-CoV-2 strains on day 28 were high and did not significantly differ from those on day 14. The median levels of inhibition of nAbs among patients on days 14 and 28 for all strains were 84.3% and 90.4%, respectively, of which, 91.1% and 93.6%, respectively, were for the group of ancestral strain and non-omicron variants, and 50.8% and 54.8% were for the omicron variants, respectively.

Over the 1-year follow-up period, the nAb levels for all strains started to decline on day 60, showing a significant decrease in levels compared to day 28. For example, delta (94% versus 88.6%, *p* = 0.008)), lambda (94.4% and 82.6%, *p* = 0.047), and delta plus (93.1% vs. 86.8%, *p* = 0.011). The median inhibition of nAbs of non-omicron variants decreased from 93.6% to 85.2%, and a similar reduction was also observed for the omicron variants, which decreased from 54.8% to 48.1%. The antibody levels against all SARS-CoV-2 strains decreased further on day 180, with median levels of inhibition of 69% for the group of ancestral strain and non-omicron variants, and 33.3% for the omicron variants (*p* = 0.002). On day 365, the median level of nAbs for the non-omicron variants was 68.1%, while that for the omicron variants was 31%. The levels of antibodies against BA.2, XBB, and XBB 1.5 variants were negative at below 30% inhibition. Due to the loss of follow-up for some patients at certain timepoints, we conducted a complete-case analysis comparing patients with and without full follow-up to assess the robustness of our findings. The nAb levels were not significantly different between these groups, as presented in Supplementary Figure S1.

### nAbs against SARS-CoV-2 variants in patients who received different vaccine regimens

**Fig. 3:**
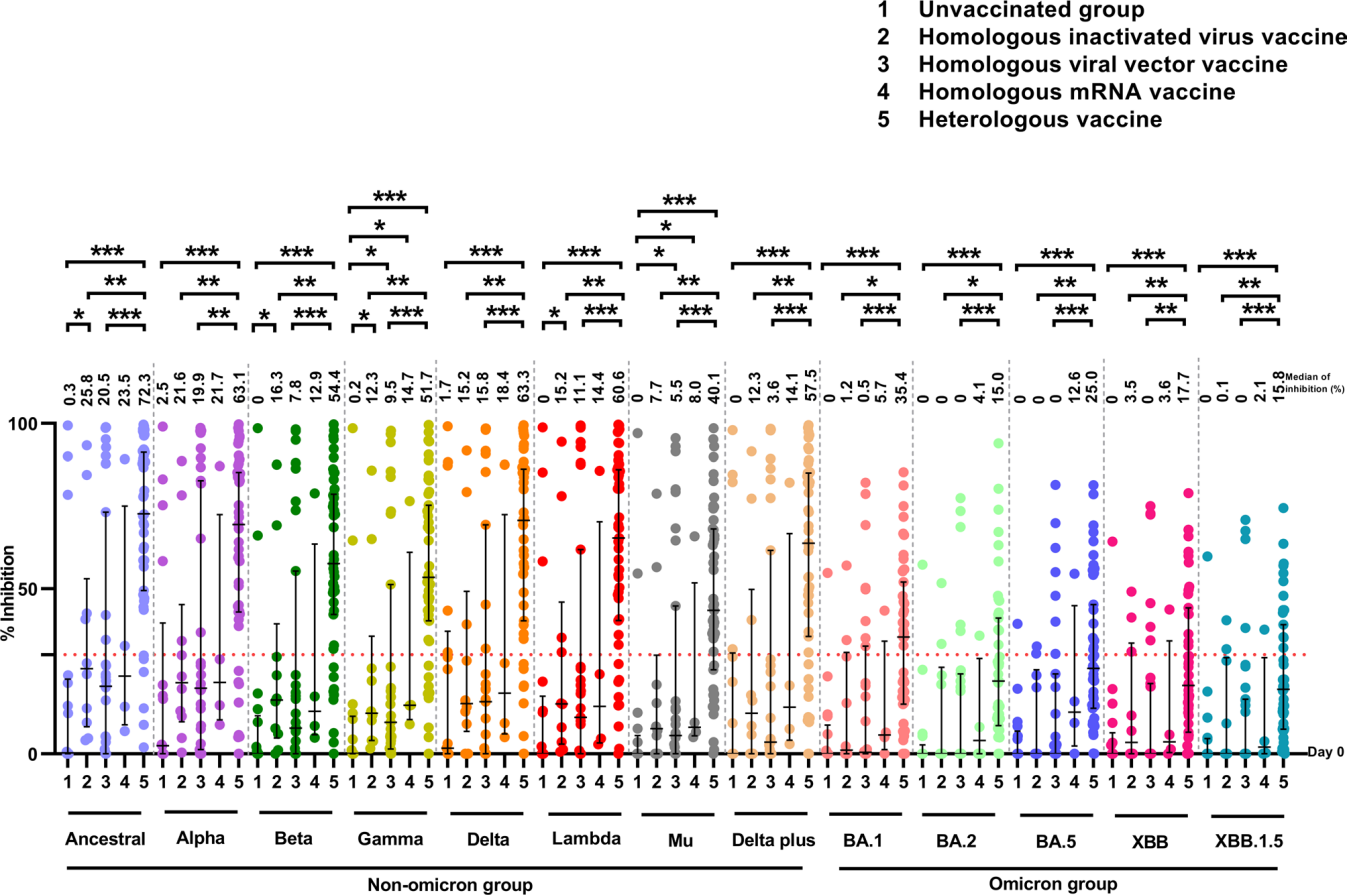
Neutralizing antibodies against the SARS-CoV-2 ancestral strain and variants on day 0 in 111 COVID-19 patients. The nAbs were determined using the multiplex surrogate virus neutralization test (sVNT) assay on plasma samples from patients who were unvaccinated (N = 17) and those who received a homologous inactivated virus vaccine (CoronaVac or BBIBP-CorV) (N = 10), homologous viral vector vaccine (ChAdOx1-S) (N = 31), homologous mRNA vaccine (BNT162b2 or mRNA-1273) (N = 4), and heterologous vaccine (all vaccine combinations) (N = 49). The Mann–Whitney U test was used to compare the median differences between groups. Numbers on the graph represent the median percent inhibition of nAbs. A percentage of inhibition value of less than 30% was considered a negative result (indicated by the dotted red line). ***, *p <* 0.001; **, p < 0.010, and *, *p <* 0.050. The bars in the graphs represent the interquartile range (IQR). Neutralizing antibody data for 111 COVID-19 patients were partially obtained from a previously published dataset^33^.

We next compared the levels of nAbs against SARS-CoV-2 ancestral strain and variants in the following groups of COVID-19 patients: group 1, unvaccinated patients; group 2, breakthrough patients with homologous inactivated virus vaccine (CoronaVac or BBIBP-CorV); group 3, breakthrough patients with a homologous viral vector vaccine (ChAdOx1-S); group 4, breakthrough patients with a homologous mRNA vaccine (BNT162b2 or mRNA-1273); and group 5, breakthrough patients with a heterologous vaccine. Breakthrough infections were identified based on patients who were confirmed by positive RT-PCR results, despite having complete a COVID-19 vaccination regimen prior to infection. The nAb responses on day 0 in all groups are shown in Figure 3 and Supplementary Table S4. The vaccine regimens received by breakthrough patients before SARS-CoV-2 infection are shown in Supplementary Table S1. Statistical comparisons of neutralizing antibody (nAb) levels against SARS-CoV-2 variants in patients receiving different vaccine regimens, using mixed-effects models, are presented in Supplementary Table S5.

The levels of nAbs against all SARS-CoV-2 strains in unvaccinated COVID-19 patients (group 1) on day 0 were low, with median and IQR inhibition values of 0% (0%–0.3%). Among all the breakthrough infection patients (groups 2–5) the levels of nAbs against the group of ancestral strain and non-omicron variants were higher than those against the omicron variants. Interestingly, we observed differences in nAb responses among patients who had previously received different vaccine regimens. Specifically, the breakthrough patients who received a heterologous vaccine (group 5) had significantly higher levels of nAbs compared to patients who received all homologous vaccines (groups 2–4) against ancestral strain, and both non-omicron and omicron variants.

**Fig. 4:**
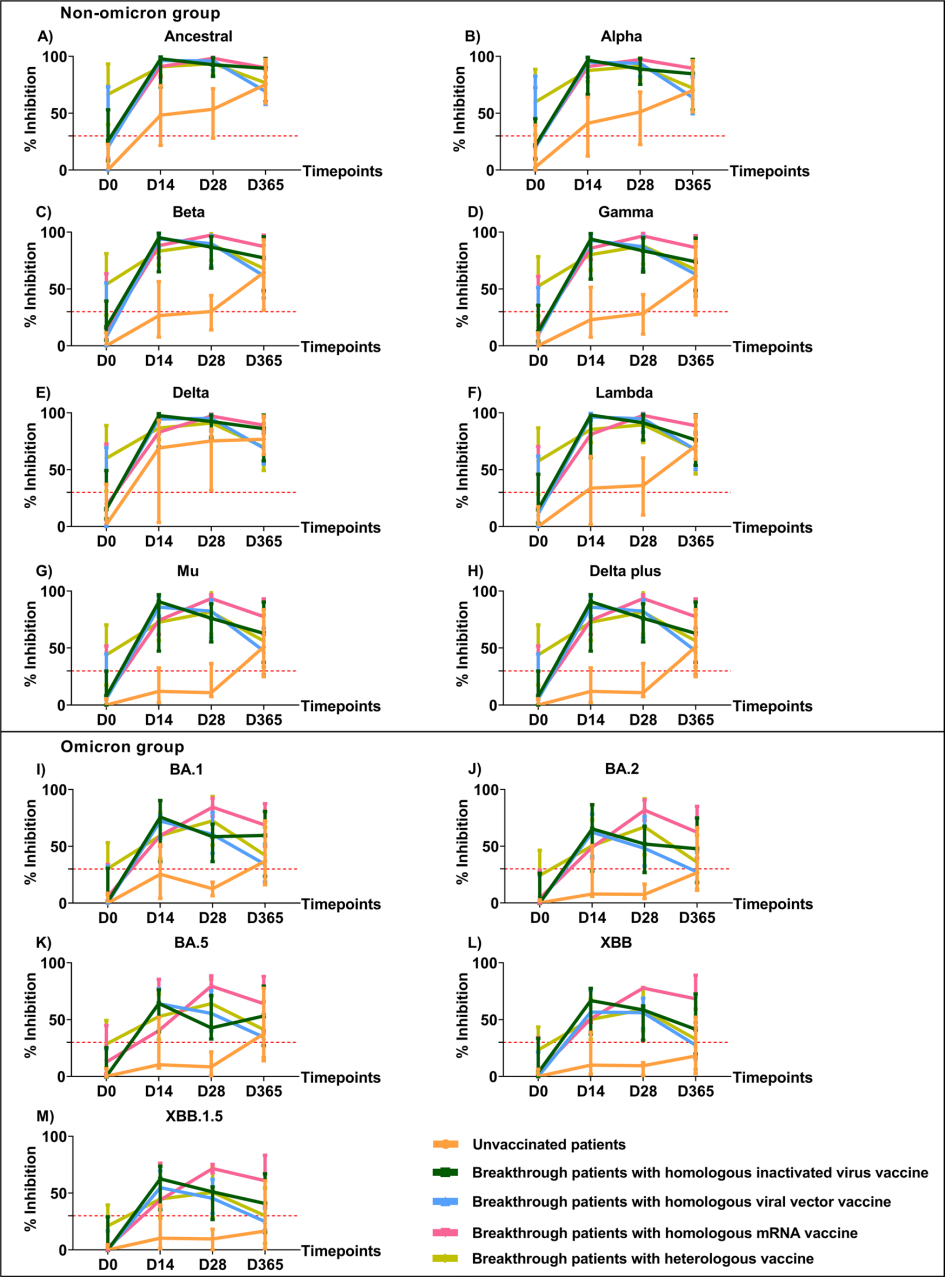
Neutralizing antibodies against the SARS-CoV-2 ancestral strain and variants in 111 COVID-19 patients who had received different vaccine regimens. The levels of nAbs against the non-omicron variants (A-H) and the omicron variants (I-M) were measured by multiplex surrogate virus neutralization test (sVNT) assay on plasma samples. Plasma was collected from COVID-19 patients who tested positive by RT-PCR. Patients consisted of groups unvaccinated patients, breakthrough patients with homologous inactivated virus vaccine (CoronaVac or BBIBP-CorV), breakthrough patients with homologous viral vector vaccine (ChAdOx1-S), breakthrough patients with homologous mRNA vaccine (BNT162b2 or mRNA-1273), and breakthrough patients with heterologous vaccine (all vaccine combinations) with group sizes of 17, 10, 31, 4, and 49, respectively (day 0); 6, 6, 16, 3, and 34, respectively (day 14); 9, 9, 22, 2, and 23, respectively (day 28); 11, 8, 15, 2, and 27, respectively (day 365). The median differences between groups were compared using a mixed-effects model. A percentage of inhibition values of less than 30% (indicated by the dotted red line) were considered negative results. ***, *p <* 0.001. The bars in the graphs represent the interquartile range (IQR). Neutralizing antibody data for 111 COVID-19 patients were partially obtained from a previously published dataset^33^.

Due to the very limited number of patients at day 60 (N = 23) and day180 (N = 20), we were unable to divide patients into all subgroups at these timepoints. The dynamic levels of nAbs in breakthrough COVID-19 patients who had received homologous (groups 2–4) and heterologous (group 5) vaccines were similar, i.e., the antibody levels significantly increased on days 14 and 28 and decreased on day 365 **(**Figure 4**, **Supplementary Table S6**)**. In contrast, the rate of increase in antibody levels in the unvaccinated group (group 1) was much slower. The levels of nAbs against the ancestral strain and non-omicron variants were higher than those against the omicron variants at all time points. The nAb levels in unvaccinated patients continued to rise, suggesting a possible subsequent infection occurring between hospital discharge and the follow-up at day 365.

### nAb levels against SARS-CoV-2 variants in COVID-19 patients with different conditions

**Fig. 5:**
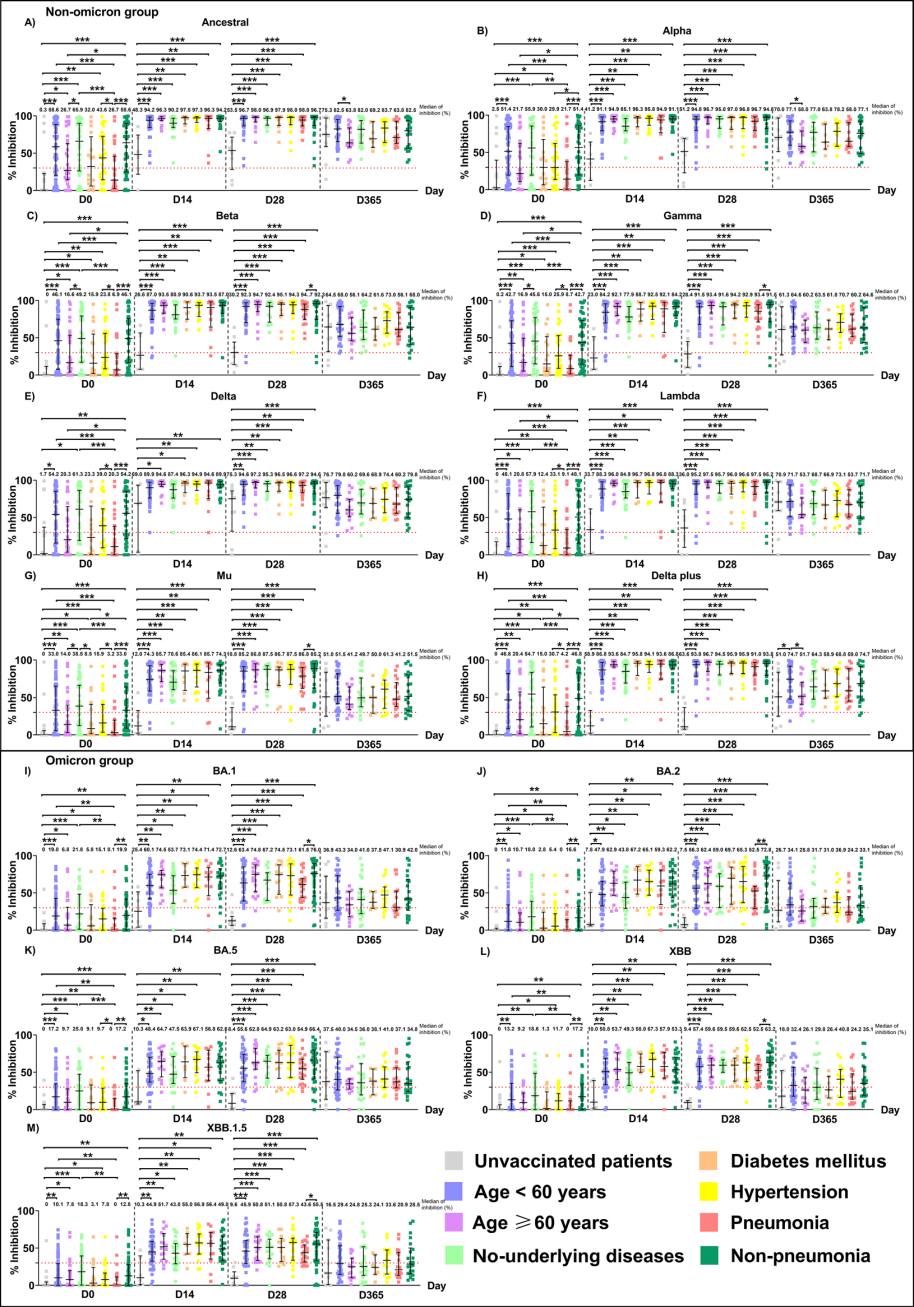
Neutralizing antibodies against the SARS-CoV-2 ancestral strain and variants in breakthrough patients with the following different conditions: unvaccinated status, aged <60 years, aged ≥60 years, no underlying diseases, diabetes mellitus, hypertension, pneumonia, and nonpneumonia. The levels of nAbs were determined by the multiplex sVNT assay on plasma samples. Patients consisted of groups unvaccinated status, age <60, age ≥60, no-underlying diseases, diabetes mellitus, hypertension, pneumonia, and nonpneumonia with group sizes of 17, 65, 29, 38, 16, 31, 28, and 44, respectively (day 0); 6, 44, 16, 28, 8, 17, 12, and 26, respectively (day 14); 9, 38, 19, 16, 13, 20, 22, and 35, respectively (day 28); 11, 39, 15, 23, 7, 16, 18, and 22, respectively (day 365). The median differences between groups were compared using the Mann-Whitney U test. Numbers on the graph represent the median percent inhibition of nAbs. A percentage of inhibition value of less than 30% was considered a negative result (indicated by the dotted red line). ***, *p <* 0.001; **, p < 0.010, and *, *p <* 0.050. The bars in the graphs represent the interquartile range (IQR). Neutralizing antibody data for 111 COVID-19 patients were partially obtained from a previously published dataset^33^.

We next evaluated the effects of age, underlying diseases, and clinical conditions on the levels of nAbs in breakthrough patients **(**Figure 5 and Supplementary Table S7-S9**)**. Patients were categorized into two age groups: <60 years (N = 65) and ≥60 years (N = 29). Vaccination, underlying diseases and clinical conditions included unvaccinated status (N = 17), diabetes mellitus (N = 16), hypertension (N = 31), pneumonia (N = 28), and nonpneumonia (N = 44). All patients with comorbidities belonged to the breakthrough group. Pneumonia was defined as clinical symptoms (fever, cough, or dyspnea) with radiographic pulmonary infiltrates. Antibody levels were evaluated at various time points in breakthrough patients, targeting all SARS-CoV-2 strains in the non-omicron and omicron variants.

Unvaccinated patients showed significantly lower nAb levels on days 0, 14, and 28 against all SARS-CoV-2 variants, compared to those with other conditions. The levels of nAbs on day 0 in breakthrough patients with pneumonia were significantly lower than those without pneumonia or with other conditions (Supplementary Table S8). We observed similar antibody responses to both the non-omicron and omicron variants. However, compared to day 0, levels of nAbs against the SARS-CoV-2 ancestral strain and variants significantly increased in all breakthrough patient groups on days 14 and 28, followed by a decrease by day 365. Notably, the levels of nAbs against the non-omicron variants were consistently higher than levels against the omicron variants in all breakthrough patient groups. Moreover, patients ≥60 years old had lower levels of nAbs on day 365 compared to patients <60 years old (Supplementary Table S9).

### Hematology measurements in unvaccinated and breakthrough COVID-19 patients

**Fig. 6:**
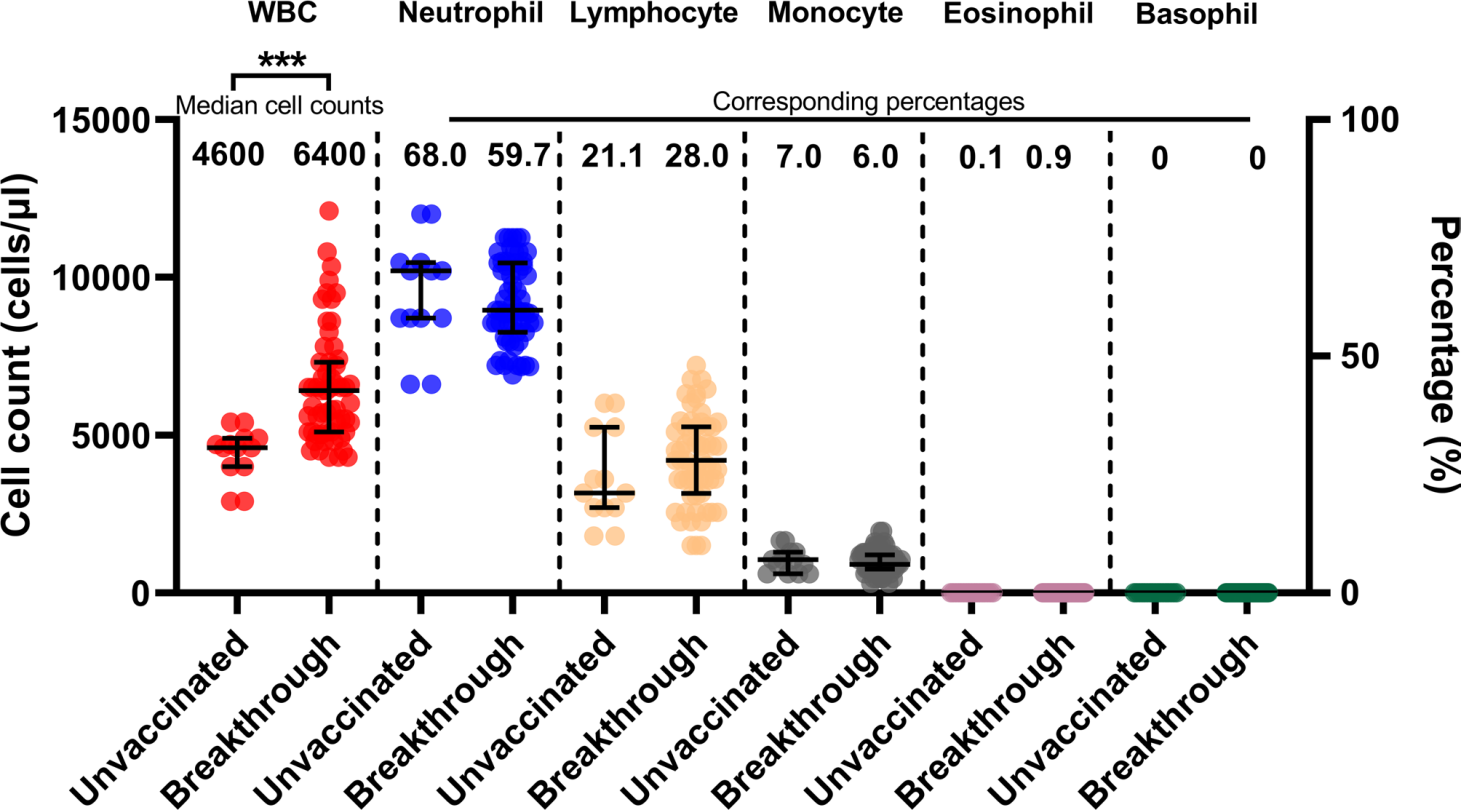
White blood cell (WBC), neutrophil, lymphocyte, monocyte, eosinophil, and basophil counts in 17 unvaccinated and 94 breakthrough COVID-19 patients on the day of hospital admission. Median differences between groups were compared using the Mann-Whitney U test. ***, *p <* 0.001. Numbers in the graph represent median cell counts and corresponding percentages. The bars in the graphs represent the interquartile range (IQR).

Comparisons of the numbers of total white blood cells (WBC), neutrophils, lymphocytes, monocytes, eosinophil, and basophil between unvaccinated and breakthrough patients **(**Figure 6 and Supplementary Table S10**)** showed that the WBC counts in breakthrough patients were significantly higher than those in unvaccinated patients (6,400 cells/µl versus 4,600 cells/µl, *p* < 0.001). Interestingly, WBC counts were associated with higher nAb levels on day 28 in breakthrough patients, whereas no such association was observed in unvaccinated individuals, as shown in Supplementary Table S11. This finding may reflect enhanced immune activation, which could contribute to more robust humoral immune responses during breakthrough infection. However, the two patient groups did not differ significantly in the numbers of neutrophils (*p* = 0.512), lymphocytes (*p* = 0.231), monocytes (*p* = 0.730), eosinophil (*p* = 0.075), and basophil (*p* = 0.233).

## Discussion

This study provides a comprehensive longitudinal analysis of nAb responses against SARS-CoV-1 and multiple SARS-CoV-2 variants in both breakthrough and unvaccinated COVID-19 patients in Thailand. Given the country’s diverse demographics, varied vaccine regimens, and extensive exposure to multiple variants, these findings are likely representative of regional populations^16,26,33,35^.

Our study revealed a lack of cross-reactivity to the spike protein of the SARS-CoV-1 in COVID-19 patients, which is in line with previous study^36^. While SARS-CoV-1 and SARS-CoV-2 are both coronaviruses that share similarities in their genes (79% genetic similarity) and infection-associated symptoms, they are distinct viruses with unique protein sequences, including their spike proteins that is the primary target of nAbs of the immune system during infection or vaccination. The spike proteins of SARS-CoV-1 and SARS-CoV-2 differ in their amino acid sequences, specifically at D614G, N501T, and N501Y in SARS-CoV-2, which contribute to differences in binding affinity and antigenic determinants^2,37,38^. The nAbs for the spike protein of SARS-CoV-2 did not bind effectively to the RBD of SARS-CoV-1 used in our sVNT assay^30,37^.

In this study, on day 14, the levels of nAbs against the group of ancestral strain and non-omicron variants were higher than those against the omicron variants, with respective medians of inhibition of 91.1% and 50.8%, respectively. This result is consistent with that in our previous study, which found that nAbs on day 14 in COVID-19 patients exhibited a 97.7% inhibition against the ancestral strain and a 32.4% inhibition against the omicron variant^16^. Another study reported that COVID-19 patients in China had higher nAb levels against the non-omicron ancestral and delta variants, with geometric mean titers (GMTs) of 392 and 304, respectively, while the GMTs against the omicron BA.1, BA.2, XBB, and XBB.1.5 variants were lower, at 212, 159, 62, and 65, respectively^39^. Reduced nAb activity against the omicron variants (as compared to that against ancestral strain and non-omicron variants) may be due to the numerus mutations in the spike protein of emerging omicron variants and their subvariants. Specifically, 39 and 31 mutations have been identified in BA.1 and BA.2 subvariants, respectively^40^. As a result, the nAb response to previous vaccinations against the ancestral strain may be less effective against the omicron variants, leading to reduced neutralizing activity. Because of this so-called COVID-19 immune imprinting or “original antigenic sin (OAS),” the initial exposure to SARS-CoV-2, either through infection or vaccination, may shape the individual’s immune response to future variants^41^. The immune system’s memory of the first encountered variant (e.g., the ancestral strain) can bias the immune response against newer variants, potentially leading to a less effective immune response as the virus evolves. In XBB.1.5 subvariants, the F486P spike protein mutation enhances infectivity by increasing positive electrostatic surface potential, resulting in higher binding affinity with the host ACE2. Consequently, compared to the wild-type, omicron and its subvariants can be more transmissible and resistant to neutralization and monoclonal antibody drugs^42,43^.

During the 2023–2024 COVID-19 vaccination campaign, the U.S. Food and Drug Administration (FDA) recommended that COVID-19 vaccines target one of the dominant XBB variants, such as XBB.1.5, XBB.1.16, or XBB.2.3 (https://www.fda.gov). Similarly, the World Health Organization’s (WHO) advisory group has recommended updating COVID-19 booster shots to target XBB subvariants (https://www.who.int). These recommendations highlight the need to adapt vaccination strategies in response to the evolving landscape of SARS-CoV-2 variants at that time. Vaccine manufacturers have developed monovalent boosters targeting XBB.1.5, which is currently the most prevalent subvariant worldwide during that period (https://investors.biontech.de). This focus on XBB-targeted vaccines align with our findings, which demonstrate reduced effectiveness of earlier vaccination regimens against omicron variants.

In addition to spike mutations, omicron variants harbor mutations in non-spike regions that can potentially confer growth advantages, higher transmission rates, and enhanced immune evasion^44^. Our findings on patients with breakthrough infection suggest that prior vaccination with the ancestral strain failed to fully induce cross-reacting antibodies against the omicron variants. This suggests that omicron variants have evolved an immune evasion strategy that may have been facilitated by COVID-19 immune imprinting. As the immune system is biased toward the original strain encountered, it may be less effective at adapting to the unique features of newer variants^41^. However, the antibody response to various viral antigens in natural infection can be more diverse and robust, resulting in the development of more durable immune memory cells^45^.

Although both vaccination and natural infection can initially enhance antibody responses, over time, levels of these antibodies and memory immune cells wane gradually. Some variants, such as delta and omicron, may partially evade the immune response, thereby reducing the effectiveness of antibodies^14,15^. The duration of antibody protection is influenced by factors such as the type and dosage of vaccine and individual variability^46^. In our study, nAb levels on day 365 were comparable to those on day 180. This persistence may be due to subsequent vaccinations received by the patients. Indeed, 46 out of 111 patients (41.4%) had received a vaccine after hospital discharge. We noted that the nAb levels in non-vaccinated patients increased over time, albeit at a slower rate compared to those in vaccinated patients. Consequently, nAbs levels in vaccinated and non-vaccinated patients did not differ on day 365, with median values of inhibition of 68.1% and 69%, respectively, for the group of ancestral and non-omicron variants, and 31% and 33.3%, respectively, for the omicron variants.

Our study revealed that nAb levels against all SARS-CoV-2 strains in unvaccinated patients were markedly lower than those in breakthrough patients. Similarly, a previous study in South Africa and Italy demonstrated that nAbs responses and cross-reactivity against ancestral, beta, delta, and omicron BA.1 variants in unvaccinated patients were poorer than those in breakthrough patients; moreover, unvaccinated patients had higher levels of pro-inflammatory markers^47,48^. The higher nAb levels in breakthrough infections compared to unvaccinated COVID-19 patients may also be attributed to the recall of immune memory and exposure to viral antigens, including nucleoprotein, from natural infection and inactivated vaccines^49^. These findings suggest the importance of vaccination in enhancing an effective immune response that is characterized by elevated nAb levels.

Our findings show that breakthrough patients who received prior heterologous vaccine regimens tended to have higher levels of nAbs on day 0 compared to those who received homologous vaccine regimens. This is consistent with previous findings that revealed consistently higher nAb levels in individuals who received a heterologous vaccine compared to those who received the homologous inactivated virus vaccine (CoronaVac or BBIBP-CorV) against SARS-CoV-2 strains^29^. The potentially enhanced immune response observed in heterologous vaccine recipients may be attributable to several factors, such as differences in antigen types, immunogenicity of the vector, vaccine interaction and synergy, as well as the genetics and underlying conditions of the patients^50–52^.

The nAb levels against the ancestral strain and omicron variants in breakthrough patients who had received BNT162b2 or mRNA-1273 vaccine tended to be higher by day 28. Previous studies have suggested that mRNA vaccines may promote efficient antigen presentation and elicit robust CD4⁺ and CD8⁺ T cell responses to SARS-CoV-2. Notably, individual who experienced breakthrough infections after BNT162b2 or mRNA-1273 vaccination also demonstrated enhanced T cell responses^53,54^. Factors such as the extended dosing interval of mRNA vaccines, their adjuvant properties, and the potential for continued affinity maturation of RBD-targeting antibodies may contribute to these responses^55–57^. The elevated responses observed in patients who received homologous mRNA regimens could reflect more consistent antigen exposure that supports rapid activation of memory B cells. Overall, our findings suggest that homologous mRNA vaccination may be associated with a more focused immune response; however, this observation should be interpreted with caution given the variability in infection history and sample size.

Among the study participants, 36.9% had pneumonia, which is a more severe clinical manifestation of COVID-19. We found that nAb levels on day 0 in breakthrough patients with pneumonia were lower and their immune response was more delayed than those without pneumonia, suggesting that the antibody production of the immune system of the former may have been impaired. A previous report found inadequate production of antibodies against SARS-CoV-2 in COVID-19 patients with pneumonia; moreover, mutations in TRNT1 (tRNA nucleotidyl transferase 1) encoding a CCA-adding enzyme, were identified in association with B cell immunodeficiency^58^. We found that nAb levels in individuals aged ≥60 declined faster by day 365 compared to those under 60 years old. Such natural decline in immune function, referred to as immunosenescence, can affect the production and maintenance of nAbs and reduce immune memory over time^59^.

We observed that higher total WBC counts were associated with nAb levels at day 28 among breakthrough patients, whereas no such association was observed in unvaccinated individuals. Although the changes in neutrophil, lymphocyte, monocyte, eosinophil, and basophil counts were not individually significant between patient groups, their cumulative effect resulted in higher total WBC counts in breakthrough patients. This may reflect a broader immune activation state contributing to enhanced antibody responses^60^. Since nAbs are produced by plasma cells derived from the lymphocyte lineage, lymphocyte counts would be expected to correlate more directly with nAb levels. Indeed, the median lymphocyte count was higher in breakthrough patients, although not significantly different from unvaccinated individuals. The observed association between total WBC counts and nAb levels on day 28 may therefore reflect the combined contribution of lymphocytes and other immune subsets^60^. Future studies with larger cohorts and detailed immune phenotyping are warranted to elucidate the potential contribution of other innate immune cell subsets to the overall antibody response.

In conclusion, our study reveals the dynamics of the immune response against SARS-CoV-2 variants among breakthrough and unvaccinated patients. The antibody responses of breakthrough patients were significantly higher than those of unvaccinated patients, indicating that vaccination bolsters immunity. However, we also identified challenges to vaccination strategies, such as omicron’s immune evasion and its limited cross-reactivity against SARS-CoV-1, which may be influenced by COVID-19 immune imprinting. Furthermore, we found that heterologous vaccines rapidly induced immune responses, suggesting their potential efficacy in a diverse population. Notably, we observed a delayed immune response in patients with pneumonia while patients ≥60 years old exhibited a faster decline in nAbs levels. These observations underline the complexity of immune responses among different demographic groups and clinical conditions. Vaccinated patients had higher WBC counts compared to the unvaccinated, suggesting that the impact of the immune function is broad, with implications beyond nAbs production. The recent recommendations by the US FDA and WHO to target XBB variants, particularly XBB.1.5, with monovalent boosters in the 2023–2024 vaccination campaign align with our findings and underscore the importance of adapting vaccination strategies to the evolving landscape of SARS-CoV-2 variants. Further research is needed to better understand the role of COVID-19 immune imprinting in shaping the immune response to emerging variants and its implications for vaccine development. Overall, our findings provide valuable insights to assist policy makers in shaping future vaccination strategies aimed at addressing the evolving landscape of SARS-CoV-2 variants and optimizing immune responses in vulnerable populations.

A limitation of this study is that, while the multiplex sVNT has been shown to correlate with live virus neutralization assays^30–34^, it measured only RBD-ACE2 blocking activity. Unlike pseudovirus assays, which provide a broader assessment of nAbs, the sVNT does not evaluate responses against other viral components, such as nucleoprotein. Moreover, pseudovirus assays typically requires BSL-3 facilities, which are impractical in our settings and were not feasible due to limited resources. Heterogeneous vaccine schedules and follow-up vaccinations limited analyses based on the interval between vaccination and infection. Another limitation is the lack of follow-up for some COVID-19 patients due to the home isolation policy announced by the Ministry of Public Health since July 2021, patient relocations, and patient inconvenience. In addition, identifying individuals with asymptomatic infections remain a challenge. None of the patients recruited in our study had received the bivalent vaccine at the time of enrollment or follow-up. Therefore, our findings do not reflect nAbs induced by updated vaccine regimens currently used in Thailand, such as those targeting against newly emerging VOCs, including BA.2.86, EG.5.1, and JN.1 variants^61^.

Further studies are needed to evaluate immune responses in individuals receiving newer vaccines, particularly in light of the potential impact of COVID-19 immune imprinting on vaccine effectiveness. This will provide valuable insights for vaccine development and inform policymakers on effective control of the current and newly emerging variants.

## Methods

### Ethics statement

This study was approved by the Ethics Committees of the Faculty of Tropical Medicine, (Approval No. MUTM 2021-028-01, dated 7 June 2021) and the Faculty of Medicine Ramathibodi Hospital (Approval No. MURA 2021/264, dated 31 March 2021), Mahidol University. The research adhered to the ethical principles outlined in the Declaration of Helsinki (2008) and the International Conference on Harmonization Good Clinical Practice guidelines. Written informed consent was obtained from all participants prior to enrollment.

### Enrollment of COVID-19 patients

In this study, we enrolled 111 COVID-19 hospitalized patients ≥18 years old and who tested positive for the SARS-CoV-2 S gene using the reverse transcription polymerase chain reaction (RT-PCR) assay^26^ (Figure 1**)**. Patients from the following three Thai hospitals were enrolled: the Hospital for Tropical Diseases, Faculty of Tropical Medicine, Mahidol University, Bangkok between July 2021 and December 2022 (N = 88); Vichaivej International Hospital, Samutsakhon Province between February 2022 and March 2022 (N = 7); and Prachathipat Hospital, Pathum Thani Province, between March 2022 and August 2022 (N = 16). Plasma samples of patients at the Hospital for Tropical Diseases were obtained on the first day of a positive RT-PCR test (referred to as day 0, N = 88) and on follow-up days 14 (N = 43), 28 (N = 65), and 365 (N = 50). Plasma samples of 23 patients at Vichaivej International Hospitals and Prachathipat Hospital were obtained on day 0 (N = 23) and on follow-up days 14 (N = 22), 60 (N = 23), 180 (N = 20), and 365 (N = 13). Clinical data were generated by the study team or obtained from the medical record. The following were the exclusion criteria: pregnancy or delivery in the previous 9 months, use of immuno-modifying, anti-inflammatory, and cell depletion biological agents in the previous 4 weeks. Breakthrough infection referred to individuals who developed COVID-19 after receiving vaccination. Pneumonia cases were defined as patients with clinical symptoms (e.g., fever, cough, dyspnea) together with radiographic evidence of pulmonary infiltrates, as diagnosed by clinicians in our research team.

### Multiplex surrogate virus neutralization test (sVNT)

Multiplex surrogate virus neutralization test (sVNT) was performed on plasma samples from the patients as previously described^30,34^. Briefly, plasma samples were diluted with assay buffer containing 1% bovine serum albumin in PBS and 1M NaCl. The diluted plasma at a ratio of 1:100 was mixed with 14-plex receptor-binding domain (RBD)-conjugated MagPlex-Avidin bead mixture (Luminex, Texas, USA) (600 beads per SARS-CoV-2 variant RBD). Then, 25 µl of bead mixture was added to each well of a 96-well plate and incubated at 25°C with shaking at 200 rpm for 1 h. Following the incubation, 50 µl of 1,000 ng/ml human ACE2 receptor (huACE2)-phycoerythrin (PE) (Custom produced by GenScript, China) conjugated was added into the wells and incubated at 25°C with shaking for 1 h. The solution in the plate was then discarded, the mixture was washed and resuspended in 75 µl of assay buffer. cPass SARS-CoV-2 positive control from a GenScript kit (China) and assay buffer were used as positive and negative controls, respectively. The fluorescence intensity of the emitted fluorescence from the beads was measured using a MAGPIX^®^ system (Luminex). Data acquisition on the MAGPIX system followed the manufacturer’s recommendations, and a minimum bead count of 50 per analyte was required for analysis. The percentage of inhibition was calculated by multiplying the median fluorescence intensity of test samples by 100 and dividing by the median fluorescence intensity of negative samples.

### Hematological measurement

Whole blood samples were collected in EDTA-anticoagulated tubes and analyzed using the Mindray BC-6200 Automated Hematology Analyzer (Mindray, China) according to the manufacturer’s instructions. Briefly, blood samples were diluted with DS diluent (Mindray, China), after which the mixture was automatically combined with M-6LD Lyse (Mindray, China) to lyse red blood cells, followed by M-6FD Dye (Mindray, China) to stain nucleic acids (RNA/DNA) in white blood cells. The five major leukocyte subsets (neutrophils, lymphocytes, monocytes, eosinophils, and basophils) were analyzed in the DIFF (WBC differential) channel based on forward-scatter and side-scatter characteristics using the SF Cube technology, a three-dimensional analysis that integrates forward scatter, side scatter, and fluorescence signals generated as cells pass through the laser beam. Total white blood cell counts were reported as the sum of all particles in the WBC region. Cells were classified based on size, granularity, and staining characteristics. Absolute counts and percentages of each WBC subtype were generated, and any abnormal results flagged by the analyzer were reviewed by a trained technician at the Hematology and Clinical Microscopy Laboratory, Hospital for Tropical Diseases.

### Statistical analysis

All data were analyzed using GraphPad Prism version 8.0 and 10.2.3 (GraphPad Software Inc., San Diego, CA, USA). The Mann–Whitney test and mixed-effects model were used to compare the median differences between groups. Spearman’s correlation was used to test the correlations between groups. *P* values of <0.05 were considered statistically significant. The bars in the graphs represent the interquartile range (IQR).

## Supplementary Information

Supplementary Information.

## Data Availability

All analyses and datasets from this study are provided in the published article and the supplementary information.
