# Supplementary material for: Longitudinal analysis of neutralizing antibodies against SARS-CoV-1 and different SARS-CoV-2 strains in breakthrough and unvaccinated COVID-19 patients in Thailand (2021-2022)
Source: Sci Rep. 2026 Jan 3;16:3408. doi: 10.1038/s41598-025-33388-7 (PMC12835167; doi:10.1038/s41598-025-33388-7)
Supplement: Supplementary file 1 — Supplementary Information. [file 41598_2025_33388_MOESM1_ESM.docx]

**Longitudinal analysis of neutralizing antibodies against SARS-CoV-1 and different SARS-CoV-2 strains in breakthrough and unvaccinated COVID-19 patients in Thailand (2021-2022)**

Prapassorn Poolchanuan^1^, Vichapon Tiacharoen^1^, Adul Dulsuk^1^, Rungnapa Phunpang^1^, Chakkaphan Runcharoen^2,3^, Thitiya Boonprakob^4^, Onura Hemtong^4^, Suchada Chowplijit^5^, Vachara Chuapaknam^5^, Tanaya Siripoon^6,7^, Watcharapong Piyaphanee^6,8^, Le Van Tan^9,10^, Susanna Dunachie^11,12^, Chee Wah Tan^13,14^, Lin-Fa Wang^13^, Wasun Chantratita^2^, Viravarn Luvira^6,7^, Narisara Chantratita^1,12^ & SEACOVARIANTS*

^1^Department of Microbiology and Immunology, Faculty of Tropical Medicine, Mahidol University, Thailand

^2^Center for Medical Genomics, Faculty of Medicine Ramathibodi Hospital, Mahidol University, Thailand

^3^Faculty of Medical Technology, Huachiew Chalermprakiet University, Thailand

^4^Prachatipat Hospital, Pathum Thani, Thailand

^5^Vichaivej International Hospital, Samut Sakhon, Thailand

^6^Department of Clinical Tropical Medicine, Faculty of Tropical Medicine, Mahidol University, Thailand

^7^Vaccine Trial Centre, Faculty of Tropical Medicine, Mahidol University, Bangkok, Thailand

^8^Thai Travel Clinic, Hospital for Tropical Diseases, Faculty of Tropical Medicine, Mahidol University, Thailand

^9^Oxford University Clinical Research Unit, Ho Chi Minh City, Vietnam

^10^Centre for Tropical Medicine and Global Health University of Oxford, United Kingdom

^11^Nuffield Department of Clinical Medicine, University of Oxford, Oxford, United Kingdom

^12^Mahidol-Oxford Tropical Medicine Research Unit, Faculty of Tropical Medicine, Mahidol University, Thailand

^13^Programme in Emerging Infectious Diseases, Duke-NUS Medical School, Singapore

^14^Infectious Diseases Translational Research Programme, Department of Microbiology and Immunology, Yong Loo Lin School of Medicine, National University of Singapore, Singapore

*A list of authors and their affiliations appears at the end of the paper.

**Corresponding author**: Narisara Chantratita, Department of Microbiology and Immunology, Faculty of Tropical Medicine, Mahidol University, 420/6 Rajvithi Road, Bangkok 10400, Thailand. E-mail: [narisara@tropmedres.ac](mailto:narisara@tropmedres.ac)

**Supplementary information**

**Supplementary Table S1** Vaccine regimens administered to COVID-19 patients before SARS-CoV-2 infection

| **Vaccine regimens** | **Number of doses** | **Number of patients^a^** |
| --- | --- | --- |
| Unvaccinated patients 0 | | 17 |
| Breakthrough infection with homologous vaccine regimens | |  |
| ChAdOx1 | 1 | 15 |
| BBIBP-CorV | 1 | 1 |
| CoronaVac + CoronaVac | 2 | 8 |
| ChAdOx1 + ChAdOx1 | 2 | 16 |
| BBIBP-CorV + BBIBP-CorV | 2 | 1 |
| BNT162b2 + BNT162b2 | 2 | 3 |
| BNT162b2 + BNT162b2 + BNT162b2 | 3 | 1 |
| Breakthrough infection with heterologous vaccine regimens | |  |
| CoronaVac + ChAdOx1 | 2 | 3 |
| CoronaVac + CoronaVac + ChAdOx1 | 3 | 6 |
| ChAdOx1 + ChAdOx1 + BNT162b2 | 3 | 13 |
| BBIBP-CorV + BBIBP-CorV + mRNA-1273 | 3 | 1 |
| BBIBP-CorV + BBIBP-CorV + BNT162b2 | 3 | 2 |
| CoronaVac + CoronaVac + ChAdOx1 + BNT162b2 | 4 | 8 |
| CoronaVac + CoronaVac + ChAdOx1 + ChAdOx1 | 4 | 2 |
| ChAdOx1 + ChAdOx1 + BNT162b2 + BNT162b2 | 4 | 3 |
| ChAdOx1 + ChAdOx1 + mRNA-1273 + mRNA-1273 | 4 | 1 |
| ChAdOx1 + ChAdOx1 + BNT162b2 + mRNA-1273 | 4 | 2 |
| CoronaVac + CoronaVac + ChAdOx1 + mRNA-1273 | 4 | 1 |
| CoronaVac + CoronaVac + BNT162b2 + mRNA-1273 | 4 | 2 |
| BBIBP-CorV + BBIBP-CorV + BNT162b2 + BNT162b2 | 4 | 1 |
| CoronaVac + CoronaVac + BNT162b2 + BNT162b2 | 4 | 1 |
| CoronaVac + CoronaVac + ChAdOx1 + BNT162b2 + BNT162b2 | 5 | 2 |
| CoronaVac + CoronaVac + ChAdOx1 + BNT162b2 + mRNA-1273 | 5 | 1 |
| Total |  | 111 |

CoronaVac and BBIBP-CorV are inactivated virus vaccine.
ChAdOx1 is a viral vector vaccine.
BNT162b2 and mRNA-1273 are mRNA vaccine.

^a^ Neutralizing antibody data for 111 COVID-19 patients were partially obtained from a previously published dataset ^33^.

| Strains | Median (IQR) percent inhibition of nAbs against SARS-CoV-1 and SARS-CoV-2 | | | | | |
| --- | --- | --- | --- | --- | --- | --- |
|  | Day 0 | Day 14 | Day 28 | Day 60 | Day 180 | Day 365 |
|  | (N = 111) | (N = 65) | (N = 65) | (N = 23) | (N = 20) | (N = 63) |
| SARS-CoV-1 | 0 (0 – 8.5) | 15.6 (5.3 – 33.5) | 17.5 (7.0 – 27.1) | 9.2 (3.6 – 21.8) | 8.0 (0.8 – 11.9) | 6.9 (2.1 – 14.3) |
| SARS-CoV-2 | | | | | | |
| Ancestral (Wuhan) | 42.6 (8.8 – 86.7) | 94.9 (84.2 – 98.3) | 96.1 (81.8 – 98.8) | 94.1 (77.2 – 96.0) | 78.2 (64.4 – 93.0) | 75.4 (61.4 – 93.9) |
| Alpha (B.1.1.7) | 38.7 (8.8 – 82.7) | 91.4 (77.8 – 97.6) | 94.1 (73.7 – 98.1) | 91.0 (68.7 – 94.4) | 72.8 (57.0 – 90.8) | 71.2 (55.7 – 91.2) |
| Beta (B.1.351) | 23.3 (3.4 – 69.2) | 86.8 (72.6 – 95.9) | 91.6 (67.2 – 97.1) | 83.6 (65.9 – 94.2) | 68.2 (49.7 – 89.6) | 64.3 (47.8 – 89.0) |
| Gamma (P.1) | 23.2 (5.0 – 65.0) | 84.3 (69.0 – 94.7) | 90.4 (62.1 – 96.4) | 79.9 (62.7 – 92.9) | 64.7 (46.1 – 87.8) | 63.0 (48.1 – 87.5) |
| Delta (B.1.617.2) | 34.8 (5.0 – 83.8) | 92.7 (77.1 – 97.5) | 94.0 (80.1 – 98.2) | 88.6 (66.5 – 94.4) | 72.1 (54.5 – 90.0) | 74.2 (55.3 – 91.7) |
| Lambda (C.37) | 26.9 (2.2 – 78.1) | 90.9 (75.4 – 97.9) | 94.4 (74.3 – 98.8) | 82.6 (64.9 – 95.4) | 69.8 (43.2 – 91.1) | 69.0 (50.8 – 88.9) |
| Mu (B.1.621) | 15.9 (0.3 – 56.5) | 75.1 (57.1 – 92.3) | 83.1 (54.1 – 91.4) | 71.8 (52.9 – 87.6) | 49.9 (32.3 – 80.6) | 50.6 (36.1 – 77.8) |
| Delta plus (AY.1) | 27.1 (0 – 77.5) | 91.3 (74.2 – 96.9) | 93.1 (74.9 – 98.1) | 86.8 (60.1 – 93.1) | 67.9 (47.0 – 87.7) | 67.1 (47.0 – 88.9) |
| BA.1 | 12.0 (0 – 37.5) | 59.9 (42.2 – 75.5) | 62.6 (38.1 – 83.9) | 58.3 (27.4 – 70.5) | 43.0 (22.7 – 55.9) | 41.0 (23.2 – 65.5) |
| BA.2 | 8.1 (0 – 25.4) | 49.7 (32.8 – 68.7) | 54.8 (26.8 – 78.9) | 49.8 (21.8 – 58.9) | 31.9 (16.9 – 49.7) | 31.0 (18.2 – 56.0) |
| BA.5 | 10.8 (0 – 30.4) | 53.6 (36.9 – 70.2) | 55.4 (33.0 – 74.3) | 48.1 (29.3 – 73.6) | 33.2 (13.8 – 52.3) | 37.5 (24.7 – 57.9) |
| XBB | 9.2 (0 – 29.2) | 50.8 (34.1 – 68.6) | 53.7 (33.4 – 67.2) | 37.5 (32.8 – 64.1) | 36.5 (16.6 – 51.8) | 27.5 (15.4 – 52.8) |
| XBB.1.5 | 7.5 (0 – 22.5) | 45.8 (32.2 – 58.6) | 44.5 (27.6 – 59.4) | 36.1 (28.3 – 54.1) | 33.3 (14.7 – 43.0) | 25.3 (13.5 – 49.5) |

**Supplementary Table S2** Neutralizing antibodies (nAbs) against SARS-CoV-1 and SARS-CoV-2 variants over one year in COVID-19 patients

^*^ Neutralizing antibody data for 111 COVID-19 patients were partially obtained from a previously published dataset^33^.

**Supplementary Table S3** Results of mixed-effects model analysis of longitudinal nAb responses against SARS-CoV-1 and SARS-CoV-2 strains in COVID-19 patients

| Effect | Numerator df | Denominator df | *P* value | Interpretation |
| --- | --- | --- | --- | --- |
| Time | 5 | 3190 | <0.001 | Significant |
| Virus | 13 | 1540 | <0.001 | Significant |
| Time × Virus | 65 | 3190 | <0.001 | Significant |

**
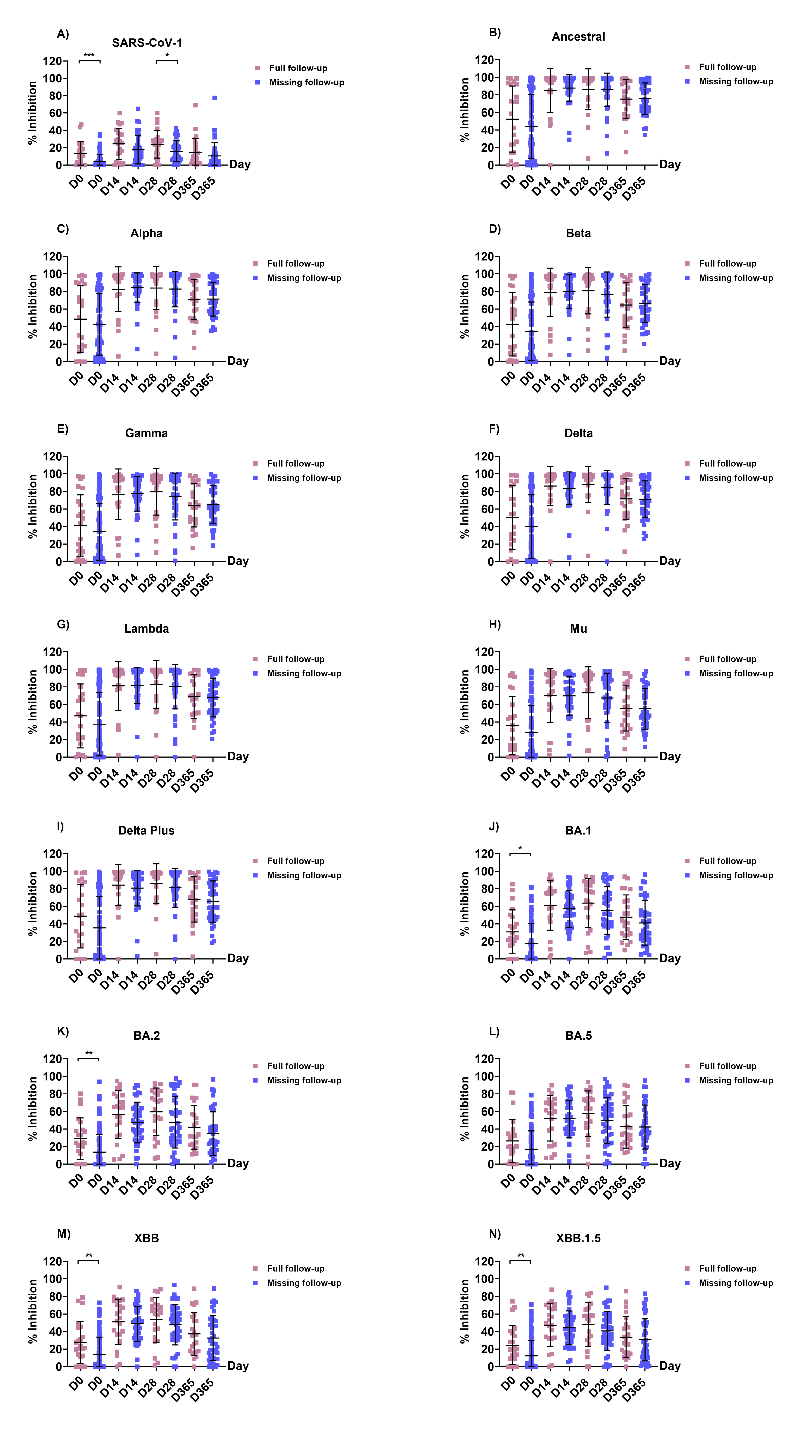
**

**Supplementary Figure S1** **Neutralizing antibodies (nAbs) against SARS-CoV-1, SARS-CoV-2 ancestral strain, and its variants over 1 year in COVID-19 patients with and without full follow-up.** nAbs were measured in plasma using the multiplex surrogate virus neutralization test (sVNT) on days 0 (N = 23 and 88), 14 (N = 23 and 42), 28 (N = 23 and 42), and 365 (N = 23 and 40) after enrollment. Median differences between groups were compared using the Mann–Whitney U test. ***, *p* < 0.001; **, *p* < 0.010; *, *p* < 0.050. Bars represent the interquartile range (IQR). nAb data for 111 patients were partially obtained from a previously published dataset^33^.

**Supplementary Table S4** Neutralizing antibodies (nAbs) against SARS-CoV-2 variants in unvaccinated patients and breakthrough patients with different vaccine regimens

| SARS-CoV-2 strain | Median (IQR) percent inhibition of nAbs against SARS-CoV-2 variants | | | | | | | | | |
| --- | --- | --- | --- | --- | --- | --- | --- | --- | --- | --- |
|  | Day 0 | | | | | Day 14 | | | | |
|  | Unvaccinated  patients | Homologous  inactivated | Homologous  viral vector | Homologous  mRNA | Heterologous | Unvaccinated  patients | Homologous  inactivated | Homologous  viral vector | Homologous  mRNA | Heterologous |
| Ancestral | 0.3 (0– 22.7) | 25.8 (8.2– 53.0) | 20.5 (0– 73.2) | 23.5 (10.2– 73.5) | 72.3 (48.0– 89.2) | 48.3 (21.4 – 74.3) | 97.9 (73.1 – 99.5) | 96.3 (95.0 – 99.3) | 91.0 (81.8 – 98.9) | 93.8 (84.7 – 97.5) |
| Alpha | 2.5 (0– 39.6) | 21.6 (9.7– 45.2) | 19.9 (1.3– 82.7) | 21.7 (10.3– 72.5) | 63.1 (42.6– 84.8) | 41.2 (12.3 – 64.0) | 96.7 (66.5 – 99.2) | 94.8 (91.6 – 98.9) | 91.0 (81.8 – 98.5) | 90.5 (80.1 – 96.4) |
| Beta | 0 (0– 11.6) | 16.3 (4.8– 39.3) | 7.8 (0– 55.4) | 12.9 (5.8– 63.5) | 54.4 (33.1– 75.5) | 26.6 (7.7 – 56.5) | 95.2 (65.2 – 99.0) | 94.1 (86.6 – 97.5) | 88.1 (77.4 – 98.6) | 83.5 (74.4 – 94.6) |
| Gamma | 0.2 (0– 11.4) | 12.3 (4.0– 35.7) | 9.5 (1.5– 51.3) | 14.7 (10.3– 61.1) | 51.7 (35.5– 73.3) | 23.0 (7.6 – 51.4) | 93.8 (58.6 – 98.7) | 92.8 (84.0 – 96.9) | 85.8 (75.9 – 98.3) | 80.1 (71.1 – 92.3) |
| Delta | 1.7 (0– 37.1) | 15.2 (6.8– 49.2) | 15.8 (0– 69.3) | 18.4 (6.1– 72.5) | 63.3 (39.3– 85.5) | 69.0 (3.6 – 93.5) | 97.5 (70.1 – 99.2) | 94.6 (91.9 – 98.8) | 82.9 (81.8 – 98.5) | 88.8 (74.7 – 96.3) |
| Lambda | 0 (0– 17.5) | 15.2 (3.0– 45.9) | 11.1 (0– 61.9) | 14.4 (3.3– 70.3) | 60.6 (36.5– 83.2) | 33.7 (1.7 – 61.6) | 98.0 (60.7 – 99.5) | 96.4 (93.6 – 99.5) | 81.0 (80.7 – 99.3) | 87.4 (76.4 – 96.8) |
| Mu | 0 (0– 5.6) | 7.7 (0– 29.9) | 5.5 (0– 44.8) | 8.0 (5.4– 51.8) | 40.1 (21.0– 64.9) | 12.0 (2.1 – 32.7) | 90.7 (47.3 – 96.9) | 85.9 (77.8 – 95.1) | 74.3 (61.9 – 95.5) | 72.2 (59.7 – 85.8) |
| Delta plus | 0 (0– 30.5) | 12.3 (0– 49.8) | 3.6 (0– 61.6) | 14.1 (4.1– 66.8) | 57.5 (31.9– 84.0) | 58.9 (2.4 – 91.9) | 96.9 (67.8 – 99.3) | 93.4 (90.5 – 98.7) | 82.1 (79.0 – 98.3) | 85.3 (72.8 – 95.5) |
| BA.1 | 0 (0– 8.7) | 1.2 (0– 30.7) | 0.5 (0– 32.6) | 5.7 (1.3– 34.2) | 35.4 (15.1– 52.0) | 25.4 (4.1 – 51.5) | 75.8 (36.4 – 90.4) | 72.3 (57.1 – 77.7) | 59.4 (49.9 – 90.1) | 59.6 (41.6 – 74.2) |
| BA.2 | 0 (0 – 2.7) | 0 (0– 26.2) | 0 (0– 24.2) | 4.1 (0– 28.9) | 15.0 (7.9– 37.6) | 7.8 (5.8 – 50.6) | 65.3 (28.2 – 86.4) | 62.3 (41.0 – 78.2) | 49.0 (39.5 – 86.7) | 47.9 (33.4 – 65.4) |
| BA.5 | 0 (0 – 6.9) | 0 (0– 25.5) | 0 (0– 24.2) | 12.6 (2.3– 44.9) | 25.0 (11.6– 38.3) | 10.3 (7.4 – 52.1) | 64.2 (39.2 – 76.1) | 64.0 (39.4 – 77.3) | 40.3 (40.0 – 85.4) | 49.5 (36.5 – 70.1) |
| XBB | 0 (0 – 6.4) | 3.5 (0– 33.5) | 0 (0– 21.3) | 3.6 (0.4– 34.2) | 17.7 (5.9– 35.7) | 10.0 (0 – 38.9) | 66.7 (37.4 – 77.4) | 56.6 (38.7 – 76.1) | 50.8 (47.6 – 76.9) | 51.3 (32.5 – 64.5) |
| XBB.1.5 | 0 (0 – 4.6) | 0.1 (0– 29.1) | 0 (0– 16.6) | 2.1 (0.1– 29.2) | 15.8 (7.3– 32.7) | 10.3 (0.4 – 39.5) | 62.5 (35.3 – 73.7) | 54.8 (35.3 – 69.7) | 43.9 (38.4 – 76.4) | 44.5 (31.6 – 56.4) |
| SARS-CoV-2 strain | Day 28 | | | | | Day 365 | | | | |
|  | Unvaccinated  patients | Homologous  inactivated | Homologous  viral vector | Homologous  mRNA | Heterologous | Unvaccinated  patients | Homologous  inactivated | Homologous  viral vector | Homologous  mRNA | Heterologous |
| Ancestral | 53.5 (28.1 – 71.5) | 92.7 (82.5 – 98.8) | 96.1 (87.6 – 98.8) | 98.3 (97.4 – 99.2) | 98.1 (93.8 – 99.3) | 75.3 (58.9 – 97.2) | 89.5 (60.1 – 98.1) | 69.2 (57.7 – 88.2) | 90.0 (81.8 – 98.2) | 74.6 (61.6 – 93.8) |
| Alpha | 51.2 (22.5 – 68.7) | 88.8 (75.4 – 98.3) | 93.9 (82.2 – 98.1) | 97.3 (95.5 – 99.1) | 97.4 (91.7 – 99.0) | 68.8 (55.0 – 95.9) | 84.7 (52.3 – 97.3) | 63.8 (49.5 – 82.5) | 89.5 (81.8 – 97.2) | 69.6 (56.6 – 90.4) |
| Beta | 30.2 (14.0 – 44.2) | 86.8 (68.3 – 96.1) | 89.9 (70.4 – 97.1) | 97.4 (95.7 – 99.1) | 96.2 (88.9 – 98.0) | 64.5 (31.3 – 93.2) | 77.3 (48.7 – 95.9) | 61.6 (42.0 – 77.7) | 87.4 (77.4 – 97.4) | 64.2 (52.3 – 90.5) |
| Gamma | 28.4 (10.3 – 45.0) | 83.5 (64.9 – 95.2) | 87.2 (67.5 – 96.3) | 96.7 (94.4 – 98.9) | 95.5 (87.4 – 97.7) | 61.3 (27.1 – 91.6) | 73.9 (48.8 – 94.8) | 62.9 (43.4 – 75.7) | 86.4 (75.9 – 96.9) | 61.8 (53.6 – 90.4) |
| Delta | 75.3 (31.7 – 85.8) | 92.3 (78.0 – 98.1) | 95.0 (84.1 – 98.4) | 97.1 (95.3 – 98.9) | 96.7 (87.0 – 98.9) | 76.7 (63.4 – 97.2) | 86.1 (57.7 – 98.0) | 68.9 (54.8 – 83.8) | 89.1 (81.8 – 96.3) | 68.0 (51.3 – 89.0) |
| Lambda | 36.0 (10.0 – 60.3) | 91.2 (76.0 – 98.5) | 94.7 (82.9 – 99.0) | 97.7 (95.8 – 99.5) | 97.5 (85.0 – 99.1) | 70.9 (59.2 – 97.8) | 76.0 (53.7 – 98.2) | 67.4 (50.0 – 82.8) | 88.7 (81.0 – 96.3) | 64.8 (50.2 – 88.9) |
| Mu | 10.8 (7.5 – 36.6) | 76.1 (55.3 – 88.8) | 82.4 (58.9 – 92.3) | 93.5 (90.1 – 96.9) | 89.6 (78.1 – 95.5) | 51.0 (25.0 – 83.7) | 62.8 (37.6 – 90.4) | 47.3 (26.7 – 67.5) | 77.5 (61.9 – 93.1) | 49.7 (37.7 – 79.5) |
| Delta plus | 63.6 (13.7 – 78.3) | 91.2 (74.1 – 98.0) | 94.2 (80.1 – 98.2) | 96.7 (94.4 – 98.9) | 95.9 (82.7 – 98.8) | 74.1 (56.5 – 96.4) | 82.3 (51.3 – 98.0) | 58.9 (49.2 – 81.8) | 88.6 (82.1 – 95.0) | 59.1 (44.0 – 86.7) |
| BA.1 | 12.6 (6.4 – 18.5) | 58.5 (36.5 – 69.5) | 60.4 (43.6 – 79.8) | 84.5 (76.5 – 92.4) | 76.0 (60.4 – 91.4) | 36.9 (16.1 – 72.4) | 59.6 (23.7 – 80.7) | 34.1 (18.6 – 52.0) | 68.7 (49.9 – 87.5) | 37.5 (23.2 – 66.8) |
| BA.2 | 7.5 (3.8 – 16.7) | 51.9 (26.8 – 67.7) | 48.0 (32.6 – 76.0) | 81.8 (72.8 – 90.7) | 71.4 (53.1 – 89.4) | 26.7 (11.2 – 66.4) | 47.0 (18.0 – 74.9) | 27.3 (18.2 – 44.8) | 62.3 (39.5 – 85.1) | 31.0 (16.9 – 56.0) |
| BA.5 | 8.4 (0.4 – 21.6) | 42.7 (33.0 – 71.3) | 55.5 (39.9 – 75.5) | 79.5 (70.4 – 88.5) | 63.5 (48.9 – 82.3) | 37.5 (13.8 – 77.6) | 53.2 (27.1 – 79.4) | 34.5 (27.4 – 54.5) | 64.0 (40.0 – 87.9) | 36.1 (22.8 – 56.5) |
| XBB | 9.4 (0.8 – 12.2) | 58.5 (32.0 – 62.0) | 56.1 (42.0 – 68.9) | 77.7 (77.0 – 78.4) | 58.9 (42.2 – 70.4) | 18.0 (2.4 – 52.1) | 41.4 (19.8 – 72.5) | 27.5 (15.4 – 43.0) | 68.3 (47.6 – 89.0) | 23.1 (11.3 – 55.5) |
| XBB.1.5 | 9.6 (0 – 18.2) | 51.1 (26.7 – 55.4) | 45.4 (32.0 – 62.1) | 71.6 (67.9 – 75.3) | 54.1 (34.2 – 67.5) | 16.5 (0.9 – 60.9) | 40.9 (15.2 – 66.9) | 24.9 (17.4 – 36.6) | 60.9 (38.4 – 83.3) | 20.8 (10.3 – 51.7) |

^*^ Neutralizing antibody data for 111 COVID-19 patients were partially obtained from a previously published dataset^33^.

**Supplementary Table S5** Results of mixed-effects model analysis of nAb responses against SARS-CoV-2 strains in COVID-19 patients receiving different vaccine regimens

| Effect | Numerator df | Denominator df | *P* value | Interpretation |
| --- | --- | --- | --- | --- |
| Time | 3 | 115 | <0.001 | Significant |
| Vaccine regimens | 4 | 106 | <0.001 | Significant |
| Time × Virus | 12 | 177 | <0.001 | Significant |

**Supplementary Table S6** Correlation between vaccine regimens and neutralizing antibody levels in COVID-19 patients

| Variant | Vaccine regimen | *p*-value of nAb levels against SARS-CoV-2 variants in COVID-19 patients | | | |
| --- | --- | --- | --- | --- | --- |
|  |  | Day 0 | Day 14 | Day 28 | Day 365 |
|  |  | *p*-value | *p*-value | *p*-value | *p*-value |
| Ancestral | Inactivated virus vs. Viral vector | >0.999 | >0.999 | >0.999 | >0.999 |
|  | Inactivated virus vs. mRNA | >0.999 | >0.999 | >0.999 | >0.999 |
|  | Inactivated virus vs. Heterologous | 0.085 | >0.999 | >0.999 | >0.999 |
|  | Viral vector vs. mRNA | >0.999 | >0.999 | >0.999 | >0.999 |
|  | Viral vector vs. Heterologous | 0.001 | 0.539 | >0.999 | >0.999 |
|  | mRNA vs. Heterologous | 0.641 | >0.999 | >0.999 | >0.999 |
| Alpha | Inactivated virus vs. Viral vector | >0.999 | >0.999 | >0.999 | 0.888 |
|  | Inactivated virus vs. mRNA | >0.999 | >0.999 | >0.999 | >0.999 |
|  | Inactivated virus vs. Heterologous | 0.118 | >0.999 | >0.999 | >0.999 |
|  | Viral vector vs. mRNA | >0.999 | >0.999 | >0.999 | 0.625 |
|  | Viral vector vs. Heterologous | 0.021 | 0.403 | >0.999 | >0.999 |
|  | mRNA vs. Heterologous | >0.999 | >0.999 | >0.999 | >0.999 |
| Beta | Inactivated virus vs. Viral vector | >0.999 | >0.999 | >0.999 | 0.742 |
|  | Inactivated virus vs. mRNA | >0.999 | >0.999 | 0.734 | >0.999 |
|  | Inactivated virus vs. Heterologous | 0.098 | >0.999 | 0.642 | >0.999 |
|  | Viral vector vs. mRNA | >0.999 | >0.999 | 0.925 | 0.486 |
|  | Viral vector vs. Heterologous | 0.001 | 0.334 | 0.652 | >0.999 |
|  | mRNA vs. Heterologous | 0.948 | >0.999 | >0.999 | >0.999 |
| Gamma | Inactivated virus vs. Viral vector | >0.999 | >0.999 | >0.999 | 0.955 |
|  | Inactivated virus vs. mRNA | >0.999 | >0.999 | 0.688 | >0.999 |
|  | Inactivated virus vs. Heterologous | 0.074 | >0.999 | 0.613 | >0.999 |
|  | Viral vector vs. mRNA | >0.999 | >0.999 | 0.900 | 0.480 |
|  | Viral vector vs. Heterologous | 0.001 | 0.279 | 0.677 | >0.999 |
|  | mRNA vs. Heterologous | >0.999 | >0.999 | >0.999 | >0.999 |
| Delta | Inactivated virus vs. Viral vector | >0.999 | >0.999 | >0.999 | 0.800 |
|  | Inactivated virus vs. mRNA | >0.999 | >0.999 | >0.999 | >0.999 |
|  | Inactivated virus vs. Heterologous | 0.094 | >0.999 | >0.999 | >0.999 |
|  | Viral vector vs. mRNA | >0.999 | >0.999 | >0.999 | 0.913 |
|  | Viral vector vs. Heterologous | 0.002 | 0.233 | >0.999 | >0.999 |
|  | mRNA vs. Heterologous | >0.999 | >0.999 | >0.999 | >0.999 |
| Lambda | Inactivated virus vs. Viral vector | >0.999 | >0.999 | >0.999 | >0.999 |
|  | Inactivated virus vs. mRNA | >0.999 | >0.999 | >0.999 | >0.999 |
|  | Inactivated virus vs. Heterologous | 0.174 | >0.999 | >0.999 | >0.999 |
|  | Viral vector vs. mRNA | >0.999 | >0.999 | >0.999 | 0.831 |
|  | Viral vector vs. Heterologous | 0.001 | 0.117 | >0.999 | >0.999 |
|  | mRNA vs. Heterologous | 0.954 | >0.999 | >0.999 | >0.999 |
| Mu | Inactivated virus vs. Viral vector | >0.999 | >0.999 | >0.999 | 0.816 |
|  | Inactivated virus vs. mRNA | >0.999 | >0.999 | 0.495 | >0.999 |
|  | Inactivated virus vs. Heterologous | 0.059 | >0.999 | 0.605 | >0.999 |
|  | Viral vector vs. mRNA | >0.999 | >0.999 | 0.862 | 0.600 |
|  | Viral vector vs. Heterologous | 0.001 | 0.130 | >0.999 | >0.999 |
|  | mRNA vs. Heterologous | >0.999 | >0.999 | >0.999 | >0.999 |
| Delta plus | Inactivated virus vs. Viral vector | >0.999 | >0.999 | >0.999 | 0.612 |
|  | Inactivated virus vs. mRNA | >0.999 | >0.999 | >0.999 | >0.999 |
|  | Inactivated virus vs. Heterologous | 0.088 | >0.999 | >0.999 | 0.758 |
|  | Viral vector vs. mRNA | >0.999 | >0.999 | >0.999 | 0.874 |
|  | Viral vector vs. Heterologous | 0.001 | 0.248 | >0.999 | >0.999 |
|  | mRNA vs. Heterologous | >0.999 | >0.999 | >0.999 | >0.999 |
| BA.1 | Inactivated virus vs. Viral vector | >0.999 | >0.999 | >0.999 | >0.999 |
|  | Inactivated virus vs. mRNA | >0.999 | >0.999 | 0.362 | >0.999 |
|  | Inactivated virus vs. Heterologous | 0.110 | >0.999 | 0.114 | >0.999 |
|  | Viral vector vs. mRNA | >0.999 | >0.999 | 0.837 | 0.746 |
|  | Viral vector vs. Heterologous | 0.004 | >0.999 | 0.407 | >0.999 |
|  | mRNA vs. Heterologous | >0.999 | >0.999 | >0.999 | 0.971 |
| BA.2 | Inactivated virus vs. Viral vector | >0.999 | >0.999 | >0.999 | >0.999 |
|  | Inactivated virus vs. mRNA | >0.999 | >0.999 | 0.450 | >0.999 |
|  | Inactivated virus vs. Heterologous | 0.245 | >0.999 | 0.224 | >0.999 |
|  | Viral vector vs. mRNA | >0.999 | >0.999 | 0.747 | 0.742 |
|  | Viral vector vs. Heterologous | 0.006 | >0.999 | 0.357 | >0.999 |
|  | mRNA vs. Heterologous | 0.957 | >0.999 | >0.999 | 0.859 |
| BA.5 | Inactivated virus vs. Viral vector | >0.999 | >0.999 | >0.999 | >0.999 |
|  | Inactivated virus vs. mRNA | >0.999 | >0.999 | 0.470 | >0.999 |
|  | Inactivated virus vs. Heterologous | 0.035 | >0.999 | 0.770 | >0.999 |
|  | Viral vector vs. mRNA | >0.999 | >0.999 | 0.628 | >0.999 |
|  | Viral vector vs. Heterologous | 0.001 | >0.999 | 0.954 | >0.999 |
|  | mRNA vs. Heterologous | >0.999 | >0.999 | >0.999 | >0.999 |
| XBB | Inactivated virus vs. Viral vector | >0.999 | >0.999 | >0.999 | >0.999 |
|  | Inactivated virus vs. mRNA | >0.999 | >0.999 | 0.182 | >0.999 |
|  | Inactivated virus vs. Heterologous | 0.500 | >0.999 | >0.999 | >0.999 |
|  | Viral vector vs. mRNA | >0.999 | >0.999 | 0.407 | 0.527 |
|  | Viral vector vs. Heterologous | 0.010 | >0.999 | >0.999 | >0.999 |
|  | mRNA vs. Heterologous | >0.999 | >0.999 | 0.653 | 0.476 |
| XBB.1.5 | Inactivated virus vs. Viral vector | >0.999 | >0.999 | >0.999 | >0.999 |
|  | Inactivated virus vs. mRNA | >0.999 | >0.999 | 0.239 | >0.999 |
|  | Inactivated virus vs. Heterologous | 0.202 | 0.960 | >0.999 | >0.999 |
|  | Viral vector vs. mRNA | >0.999 | >0.999 | 0.383 | 0.729 |
|  | Viral vector vs. Heterologous | 0.001 | >0.999 | >0.999 | >0.999 |
|  | mRNA vs. Heterologous | >0.999 | >0.999 | 0.697 | 0.635 |

**Supplementary Table S7** Neutralizing antibodies (nAbs) against SARS-CoV-2 variants in breakthrough COVID-19 patients with different conditions

| Variants | Timepoints | Median (IQR) percent inhibition of nAbs against SARS-CoV-2 variants | | | | | | | |
| --- | --- | --- | --- | --- | --- | --- | --- | --- | --- |
|  |  | Unvaccinated patients | Aged <60 | Aged ≥ 60 | No-underlying | Diabetes | Hypertension | Pneumonia | No-pneumonia |
| Ancestral | D0 | 0.30 (0 – 22.7) | 58.6 (20.4 – 88.6) | 26.7 (11.3 – 70.1) | 65.9 (26.2 – 89.9) | 32.0 (5.9 – 72.0) | 43.6 (14.3 – 72.5) | 26.7 (11.3 – 70.1) | 58.6 (20.4 – 88.6) |
|  | D14 | 48.3 (21.7 – 74.3) | 94.2 (84.4 – 98.4) | 96.3 (94.0 – 98.7) | 90.2 (84.4 – 96.8) | 97.5 (89.3 – 98.8) | 97.3 (91.2 – 99.0) | 96.3 (94.0 – 98.7) | 94.2 (84.4 – 98.4) |
|  | D28 | 53.5 (28.1 – 71.5) | 96.7 (86.5 – 98.7) | 98.0 (91.9 – 99.0) | 96.9 (87.6 – 98.8) | 97.9 (87.0 – 98.7) | 98.0 (88.1 – 98.9) | 98.0 (91.9 – 99.0) | 96.7 (86.5 – 98.7) |
|  | D365 | 75.3 (58.9 – 97.2) | 82.5 (65.5 – 95.1) | 63.8 (58.4 – 83.9) | 82.0 (63.5 – 93.8) | 69.2 (57.7 -92.2) | 83.7 (64.1 – 92.2) | 63.8 (58.4 – 83.9) | 82.5 (65.5 – 95.1) |
| Alpha | D0 | 2.50 (0 – 39.6) | 51.4 (19.6 – 84.9) | 21.7 (11.1 – 62.3) | 55.9 (19.6 – 85.4) | 30.0 (6.4 – 67.4) | 29.9 (13.7 – 61.9) | 21.7 (11.1 – 62.3) | 51.4 (19.6 – 84.9) |
|  | D14 | 41.2 (12.3 – 64.0) | 91.1 (78.9 – 97.6) | 94.9 (91.0 – 98.3) | 85.1 (79.0 – 95.5) | 96.3 (84.3 – 98.3) | 95.8 (86.5 – 98.7) | 94.9 (91.0 – 98.3) | 91.1 (78.9 – 97.6) |
|  | D28 | 51.2 (22.5 – 68.7) | 94.8 (79.7 – 98.1) | 96.7 (88.3 – 98.4) | 95.0 (82.3 – 98.3) | 97.0 (81.0 – 97.9) | 96.8 (81.9 – 98.1) | 96.7 (88.3 – 98.4) | 94.8 (79.7 – 98.1) |
|  | D365 | 70.0 (51.0 – 96.2) | 77.1 (59.6 – 92.3) | 58.0 (50.7 – 78.7) | 77.0 (50.7 – 90.4) | 63.8 (54.4 – 88.1) | 78.2 (58.1 – 89.4) | 58.0 (50.7 – 78.7) | 77.1 (59.6 – 92.3) |
| Beta | D0 | 0 (0 – 11.6) | 46.1 (7.6 – 75.1) | 16.6 (4.0 – 53.5) | 49.2 (17.2 – 79.2) | 15.9 (2.1 – 55.0) | 23.8 (8.5 – 56.1) | 6.9 (0 – 28.6) | 46.1 (7.6 – 75.1) |
|  | D14 | 26.6 (7.7 – 56.5) | 87.0 (74.7 – 96.2) | 93.5 (82.9 – 96.9) | 80.9 (75.3 – 94.2) | 90.6 (75.0 – 98.1) | 93.7 (78.6 – 97.3) | 93.5 (82.9 – 96.9) | 87.0 (74.7 – 96.2) |
|  | D28 | 30.2 (14.0 – 44.2) | 92.3 (70.4 – 97.0) | 94.7 (85.0 – 98.3) | 92.4 (71.0 – 97.5) | 95.1 (80.0 – 97.1) | 94.3 (82.5 – 97.8) | 94.7 (85.0 – 98.3) | 92.3 (70.4 – 97.0) |
|  | D365 | 64.5 (31.3 – 93.2) | 68.0 (51.5 – 92.2) | 56.1 (45.9 – 77.0) | 64.2 (47.8 – 87.2) | 61.6 (46.9 – 77.0) | 73.0 (56.1 – 84.8) | 56.1 (45.9 – 77.0) | 68.0 (51.5 – 92.2) |
| Gamma | D0 | 0.2 (0 – 11.4) | 42.7 (11.4 – 72.9) | 16.9 (5.4 – 49.5) | 45.6 (15.0 – 77.1) | 15.0 (5.2 – 51.7) | 25.9 (8.9 – 53.0) | 8.7 (1.5 – 26.6) | 42.7 (11.4 – 72.9) |
|  | D14 | 23.0 (7.6 – 51.4) | 84.2 (69.9 – 95.6) | 92.1 (79.8 – 96.2) | 77.9 (71.5 – 91.9) | 88.7 (72.4 – 97.6) | 92.5 (74.8 – 96.6) | 92.1 (79.8 – 96.2) | 84.2 (69.9 – 95.6) |
|  | D28 | 28.4 (10.3 – 45.0) | 91.5 (68.1 – 96.3) | 93.4 (83.0 – 97.9) | 91.6 (67.2 – 96.9) | 94.2 (76.0 – 96.4) | 92.9 (77.6 – 97.4) | 93.4 (83.0 – 97.9) | 91.5 (68.1 – 96.3) |
|  | D365 | 61.3 (27.1 – 91.6) | 64.6 (51.8 – 91.4) | 60.2 (43.9 – 73.7) | 63.5 (48.2 – 84.7) | 61.8 (47.4 – 73.7) | 70.7 (57.2 – 82.2) | 60.2 (43.9 – 73.7) | 64.6 (51.8 – 91.4) |
| Delta | D0 | 1.7 (0– 37.1) | 54.2 (15.7 – 85.3) | 20.3 (3.5 – 64.7) | 61.3 (19.2 – 86.3) | 23.3 (0.4 – 65.3) | 39.0 (9.3 – 61.7) | 20.3 (3.5 – 64.7) | 54.2 (15.7 – 85.3) |
|  | D14 | 69.0 (3.6 – 93.5) | 89.9 (76.1 – 97.5) | 94.6 (91.9 – 98.3) | 87.4 (76.1 – 94.9) | 96.3 (83.3 – 98.4) | 94.9 (86.2 – 98.5) | 94.6 (91.9 – 98.3) | 89.9 (76.1 – 97.5) |
|  | D28 | 75.3 (31.7 – 85.8) | 94.6 (80.1 – 98.0) | 97.2 (89.7 – 98.4) | 95.3 (85.1 – 98.3) | 96.5 (85.1 – 97.9) | 96.6 (85.5 – 98.4) | 97.2 (89.7 – 98.4) | 94.6 (80.1 – 98.0) |
|  | D365 | 76.7 (63.4 – 97.2) | 79.8 (55.7 – 92.8) | 60.2 (51.3 – 74.6) | 69.6 (54.8 – 87.9) | 68.9 (49.3 – 89.0) | 74.4 (56.5 – 89.7) | 60.2 (51.3 – 74.6) | 79.8 (55.7 – 92.8) |
| Lambda | D0 | 0 (0– 17.5) | 48.1 (10.8 – 82.2) | 20.8 (2.8 – 60.4) | 57.9 (16.6 – 85.8) | 12.4 (1.1 – 63.9) | 33.1 (8.3 – 58.9) | 9.1 (0 – 34.1) | 48.1 (10.8 – 82.2) |
|  | D14 | 33.7 (1.7 – 61.6) | 88.3 (76.3 – 97.9) | 96.0 (92.6 – 99.0) | 84.9 (76.3 – 95.2) | 96.7 (76.2 – 99.2) | 96.8 (81.6 – 99.4) | 96.0 (92.6 – 99.0) | 88.3 (76.3 – 97.9) |
|  | D28 | 36.0 (10.0 – 60.3) | 95.2 (81.1 – 98.7) | 97.5 (89.9 – 99.1) | 95.7 (82.6 – 98.9) | 96.0 (81.5 – 98.6) | 96.6 (84.4 – 99.0) | 97.5 (89.9 – 99.1) | 95.2 (81.1 – 98.7) |
|  | D365 | 70.9 (59.2 – 97.8) | 71.7 (51.0 – 93.8) | 53.7 (50.0 – 76.4) | 68.7 (53.7 – 85.3) | 66.9 (48.5 – 86.8) | 73.1 (51.0 – 85.8) | 53.7 (50.0 – 76.4) | 71.7 (51.0 – 93.8) |
| Mu | D0 | 0 (0– 5.6) | 33.0 (5.5 – 65.3) | 14.0 (3.1 – 39.9) | 38.5 (12.4 – 66.5) | 8.5 (1.5 – 41.0) | 15.9 (3.6 – 40.9) | 3.2 (0 – 19.7) | 33.0 (5.5 – 65.3) |
|  | D14 | 12.0 (2.1 – 32.7) | 74.3 (58.8 – 92.4) | 85.7 (71.8 – 94.3) | 70.6 (60.5 – 85.7) | 85.4 (59.4 – 94.5) | 86.1 (64.1 – 94.6) | 85.7 (71.8 – 94.3) | 74.3 (58.8 – 92.4) |
|  | D28 | 10.8 (7.5 – 36.6) | 85.2 (59.0 – 91.1) | 86.8 (71.0 – 94.9) | 87.5 (60.6 – 92.4) | 86.7 (66.0 – 90.7) | 87.5 (67.6 – 93.9) | 86.8 (71.0 – 94.9) | 85.2 (59.0 – 91.1) |
|  | D365 | 51.0 (25.0 – 83.7) | 51.5 (41.9 -81.7) | 41.2 (34.0 – 64.2) | 49.7 (37.7 – 76.2) | 50.0 (29.0 – 64.2) | 61.3 (35.8 – 73.5) | 41.2 (34.0 – 64.2) | 51.5 (41.9 – 81.7) |
| Delta plus | D0 | 0 (0– 30.5) | 46.8 (8.2 – 82.0) | 20.4 (1.3 – 57.6) | 54.7 (10.5 – 83.5) | 15.0 (0 – 64.0) | 30.7 (3.0 – 53.6) | 4.2 (0 – 38.1) | 46.8 (8.2 – 82.0) |
|  | D14 | 58.9 (2.4 – 91.9) | 86.8 (74.0 – 96.9) | 93.6 (90.5 – 98.1) | 84.7 (69.4 – 94.2) | 95.8 (79.4 – 98.2) | 94.1 (83.5 – 98.2) | 93.6 (90.5 – 98.1) | 86.8 (74.0 – 96.9) |
|  | D28 | 63.6 (13.7 – 78.3) | 93.8 (78.7 – 97.9) | 96.7 (84.8 – 98.3) | 94.5 (80.3 – 98.1) | 95.9 (81.8 – 97.8) | 95.9 (82.0 – 98.3) | 91.0 (80.2 – 96.8) | 93.8 (78.7 – 97.9) |
|  | D365 | 51.0 (25.0- – 83.7) | 74.7 (47.9 – 91.8) | 51.7 (41.0 – 70.4) | 64.3 (47.9 – 85.3) | 58.9 (49.2 – 86.7) | 68.8 (50.9 – 87.9) | 59.0 (45.2 – 88.4) | 74.7 (47.9 – 91.8) |
| BA.1 | D0 | 0 (0– 8.7) | 19.0 (0.5 – 42.7) | 6.8 (0 – 30.8) | 21.8 (1.2 – 48.3) | 5.8 (0 – 34.3) | 15.1 (0 – 29.5) | 0.1 (0 – 16.1) | 19.9 (0.2 – 41.8) |
|  | D14 | 25.4 (4.1 – 51.5) | 60.1 (42.1 – 75.1) | 74.5 (56.9 – 80.3) | 53.7 (35.9 – 73.0) | 73.1 (57.6 – 89.3) | 74.4 (57.0 – 83.3) | 71.4 (46.1 – 83.7) | 72.7 (55.0 – 82.5) |
|  | D28 | 12.6 (6.4 – 18.5) | 63.4 (39.1 – 84.9) | 74.8 (51.6 – 87.5) | 67.2 (39.9 – 77.1) | 74.5 (48.2 – 87.7) | 73.1 (47.2 – 88.9) | 61.5 (43.6 – 69.0) | 76.0 (45.7 – 89.3) |
|  | D365 | 36.9 (16.1 – 72.4) | 43.3 (26.7 – 73.0) | 34.0 (18.0 – 51.7) | 41.0 (22.1 – 55.3) | 37.5 (29.4 – 51.8) | 47.1 (30.6 – 58.0) | 30.9 (25.8 – 52.4) | 42.0 (26.7 – 68.4) |
| BA.2 | D0 | 0 (0 – 2.7) | 11.8 (0 – 33.9) | 10.7 (0 – 23.1) | 18.0 (0 – 38.7) | 2.8 (0 – 24.3) | 5.4 (0 – 22.0) | 0 (0 – 14.1) | 16.6 (0 – 31.8) |
|  | D14 | 7.8 (5.8 – 50.6) | 47.9 (32.5 – 65.6) | 62.9 (46.7 – 79.4) | 43.8 (29.4 – 64.4) | 67.2 (48.4 – 85.1) | 65.1 (46.9 – 80.5) | 59.3 (34.3 – 80.7) | 62.2 (43.4 – 79.3) |
|  | D28 | 7.5 (3.8 – 16.7) | 56.3 (29.5 – 80.2) | 62.4 (36.9 – 83.9) | 59.0 (30.2 – 75.7) | 69.7 (34.9 – 84.7) | 65.3 (36.9 – 85.6) | 52.5 (29.2 – 58.7) | 72.8 (36.9 – 87.5) |
|  | D365 | 26.7 (11.2 – 66.4) | 34.1 (20.2 – 63.2) | 25.8 (12.3 – 41.5) | 31.7 (16.2 – 42.7) | 31.0 (22.5 – 42.6) | 36.9 (23.7 – 51.0) | 24.2 (20.5 – 45.0) | 33.1 (19.7 – 59.8) |
| BA.5 | D0 | 0 (0 – 6.9) | 17.2 (0 – 34.4) | 9.7 (0 – 27.6) | 25.0 (0.1 – 47.5) | 9.1 (0 – 32.9) | 9.7 (0 – 28.7) | 0 (0 – 14.5) | 17.2 (2.3 – 35.0) |
|  | D14 | 10.3 (7.4 – 52.1) | 48.4 (36.8 – 70.2) | 64.7 (43.7 – 77.3) | 47.5 (35.0 – 71.3) | 63.9 (42.7 – 85.2) | 67.1 (49.5 – 77.4) | 56.8 (38.6 – 79.4) | 62.6 (38.9 – 71.0) |
|  | D28 | 8.4 (0.4 – 21.6) | 55.6 (37.3 – 74.0) | 62.8 (54.1 – 82.0) | 64.9 (42.2 – 72.7) | 63.2 (41.7 – 79.5) | 63.0 (42.8 – 86.0) | 54.9 (40.6 – 67.5) | 66.4 (41.0 – 79.8) |
|  | D365 | 37.5 (13.8 – 77.6) | 40.0 (26.2 – 70.3) | 34.5 (27.4 – 41.1) | 36.0 (19.6 – 61.6) | 38.1 (19.7 – 54.5) | 41.0 (29.7 – 57.3) | 37.1 (29.2 – 56.0) | 34.8 (24.3 – 64.0) |
| XBB | D0 | 0 (0 – 6.4) | 13.2 (0 – 35.1) | 9.2 (0 – 22.2) | 18.6 (0 – 48.6) | 1.3 (0 – 23.8) | 11.7 (0 – 21.2) | 0 (0 – 11.6) | 17.2 (0 – 34.3) |
|  | D14 | 10.0 (0 – 38.9) | 50.8 (33.3 – 68.7) | 53.7 (39.9 – 76.1) | 49.3 (32.4 – 63.2) | 58.0 (50.0 – 74.5) | 67.3 (44.3 – 75.8) | 57.9 (42.1 – 76.5) | 53.3 (36.6 – 70.3) |
|  | D28 | 9.4 (0.8 – 12.2) | 57.4 (37.7 – 68.3) | 59.6 (43.4 – 68.8) | 59.5 (50.0 – 66.8) | 59.6 (38.1 – 69.0) | 62.5 (37.3 – 70.3) | 52.5 (38.5 – 60.4) | 63.2 (43.4 – 77.0) |
|  | D365 | 18.0 (2.4 – 52.1) | 32.4 (17.9 – 56.6) | 26.1 (8.7 – 43.0) | 29.8 (13.5 – 55.5) | 26.4 (15.4 – 44.5) | 40.8 (16.4 – 53.9) | 24.2 (13.9 – 47.9) | 35.1 (18.8 – 57.0) |
| XBB.1.5 | D0 | 0 (0 – 4.6) | 10.1 (0 – 29.2) | 7.8 (0 – 19.6) | 18.3 (0.3 – 39.4) | 3.1 (0 – 24.0) | 7.8 (0 – 16.6) | 0 (0 – 11.3) | 12.8 (0 – 28.0) |
|  | D14 | 10.3 (0.4 – 39.5) | 44.9 (32.4 – 58.8) | 51.7 (35.1 – 69.7) | 43.0 (28.9 – 56.1) | 55.0 (42.0 – 69.3) | 56.9 (42.0 – 68.9) | 56.4 (35.3 – 70.9) | 49.0 (32.9 – 65.9) |
|  | D28 | 9.6 (0 – 18.2) | 45.9 (29.4 – 60.1) | 50.8 (33.5 – 65.1) | 51.1 (35.6 – 61.8) | 50.8 (32.0 – 63.0) | 57.3 (33.7 – 63.3) | 43.6 (31.3 – 54.3) | 55.5 (32.8 – 70.8) |
|  | D365 | 16.5 (0.9 – 60.9) | 29.4 (14.8 – 53.2) | 24.8 (10.3 – 36.3) | 25.3 (13.5 – 51.7) | 24.1 (14.8 – 40.9) | 33.6 (15.1 – 47.5) | 20.9 (13.5 – 44.2) | 28.5 (15.6 – 50.1) |

^*^ Neutralizing antibody data for 111 COVID-19 patients were partially obtained from a previously published dataset^33^.

**Supplementary Table S8** Correlation between pneumonia and neutralizing antibody levels in COVID-19 patients

| Variants | Pneumonia | *p*-value of nAb levels against SARS-CoV-2 variants in COVID-19 patients | | | |
| --- | --- | --- | --- | --- | --- |
|  |  | Day 0 | Day 14 | Day 28 | Day 365 |
|  |  | *p*-value | *p*-value | *p*-value | *p*-value |
| Ancestral | Yes | 0.001 | 0.637 | 0.056 | 0.607 |
|  | No |  |  |  |  |
| Alpha | Yes | <0.001 | 0.427 | 0.067 | 0.610 |
|  | No |  |  |  |  |
| Beta | Yes | 0.001 | 0.398 | 0.026 | 0.697 |
|  | No |  |  |  |  |
| Gamma | Yes | 0.001 | 0.427 | 0.022 | 0.840 |
|  | No |  |  |  |  |
| Delta | Yes | 0.001 | 0.950 | 0.166 | 0.587 |
|  | No |  |  |  |  |
| Lambda | Yes | 0.001 | 0.720 | 0.053 | 0.675 |
|  | No |  |  |  |  |
| Mu | Yes | 0.001 | 0.500 | 0.019 | 0.859 |
|  | No |  |  |  |  |
| Delta plus | Yes | 0.001 | 0.970 | 0.116 | 0.840 |
|  | No |  |  |  |  |
| BA.1 | Yes | 0.004 | 0.605 | 0.020 | 0.541 |
|  | No |  |  |  |  |
| BA.2 | Yes | 0.010 | 0.678 | 0.008 | 0.352 |
|  | No |  |  |  |  |
| BA.5 | Yes | 0.001 | 0.715 | 0.052 | 0.825 |
|  | No |  |  |  |  |
| XBB | Yes | 0.003 | 0.816 | 0.013 | 0.383 |
|  | No |  |  |  |  |
| XBB.1.5 | Yes | 0.002 | 0.772 | 0.047 | 0.600 |
|  | No |  |  |  |  |

**Supplementary Table S9** Correlation between age and neutralizing antibody levels in COVID-19 patients

| Variants | Age | Spearman’s correlation (r) and *p*-value of nAb levels against SARS-CoV-2 variants in COVID-19 patients | | | | | | | |
| --- | --- | --- | --- | --- | --- | --- | --- | --- | --- |
|  |  | Day 0 | | Day 14 | | Day 28 | | Day 365 | |
|  |  | r | *p*-value | r | *p*-value | r | *p*-value | r | *p*-value |
| Ancestral | < 60 | 0.122 | 0.297 | 0.360 | 0.013 | 0.374 | 0.003 | 0.047 | 0.764 |
|  | ≥ 60 | -0.088 | 0.613 | 0.197 | 0.419 | 0.093 | 0.657 | 0.135 | 0.572 |
| Alpha | < 60 | 0.181 | 0.120 | 0.413 | 0.004 | 0.357 | 0.004 | 0.191 | 0.194 |
|  | ≥ 60 | 0.154 | 0.377 | 0.240 | 0.322 | 0.093 | 0.660 | 0.297 | 0.179 |
| Beta | < 60 | 0.228 | 0.053 | 0.385 | 0.016 | 0.330 | 0.009 | 0.035 | 0.811 |
|  | ≥ 60 | 0.022 | 0.901 | 0.132 | 0.611 | 0.044 | 0.836 | -0.154 | 0.494 |
| Gamma | < 60 | 0.096 | 0.418 | 0.272 | 0.094 | 0.317 | 0.012 | 0.001 | 0.996 |
|  | ≥ 60 | -0.104 | 0.551 | 0.075 | 0.773 | 0.015 | 0.942 | -0.165 | 0.463 |
| Delta | < 60 | 0.237 | 0.052 | 0.316 | 0.033 | 0.382 | 0.002 | -0.012 | 0.933 |
|  | ≥ 60 | 0.062 | 0.734 | 0.186 | 0.445 | -0.090 | 0.669 | -0.036 | 0.873 |
| Lambda | < 60 | 0.099 | 0.407 | 0.202 | 0.218 | 0.305 | 0.016 | -0.026 | 0.860 |
|  | ≥ 60 | -0.133 | 0.448 | -0.015 | 0.956 | 0.030 | 0.886 | -0.087 | 0.700 |
| Mu | < 60 | 0.040 | 0.733 | 0.298 | 0.049 | 0.331 | 0.014 | 0.155 | 0.282 |
|  | ≥ 60 | -0.128 | 0.462 | 0.080 | 0.744 | -0.163 | 0.457 | 0.262 | 0.239 |
| Delta plus | < 60 | 0.190 | 0.103 | 0.376 | 0.010 | 0.343 | 0.006 | 0.144 | 0.329 |
|  | ≥ 60 | 0.029 | 0.868 | 0.261 | 0.281 | 0.036 | 0.865 | 0.280 | 0.208 |
| BA.1 | < 60 | 0.230 | 0.059 | 0.059 | 0.698 | 0.181 | 0.159 | -0.115 | 0.420 |
|  | ≥ 60 | -0.035 | 0.846 | 0.253 | 0.295 | -0.277 | 0.180 | -0.260 | 0.243 |
| BA.2 | < 60 | 0.068 | 0.560 | 0.281 | 0.056 | 0.354 | 0.006 | 0.125 | 0.426 |
|  | ≥ 60 | -0.242 | 0.161 | 0.249 | 0.304 | 0.002 | 0.994 | 0.095 | 0.690 |
| BA.5 | < 60 | 0.026 | 0.822 | 0.132 | 0.394 | 0.184 | 0.180 | -0.093 | 0.523 |
|  | ≥ 60 | -0.153 | 0.379 | 0.101 | 0.682 | -0.069 | 0.755 | 0.285 | 0.198 |
| XBB | < 60 | 0.053 | 0.647 | 0.140 | 0.366 | 0.158 | 0.249 | 0.036 | 0.803 |
|  | ≥ 60 | -0.280 | 0.103 | 0.084 | 0.733 | 0.061 | 0.784 | 0.179 | 0.425 |
| XBB.1.5 | < 60 | 0.320 | 0.008 | 0.332 | 0.024 | 0.263 | 0.037^*^ | 0.034 | 0.814 |
|  | ≥ 60 | 0.001 | 0.994 | 0.378 | 0.111 | 0.188 | 0.368 | -0.093 | 0.680 |

**Supplementary Table S10** Median (IQR) number of white blood cells and percentage of neutrophil, lymphocyte, and monocyte counts in unvaccinated COVID-19 patients and breakthrough COVID-19 patients on the hospital admission day.

| Type of cells | Vaccinated conditions | | |
| --- | --- | --- | --- |
|  | Unvaccinated | Breakthrough | *P*-Value |
| White blood cell (cell/µl) | 4600 (4000 – 4900) | 6400 (5100 – 7300) | 0.001 |
| Neutrophil (%) | 68.0 (58.0 – 69.8) | 59.7 (55.0 – 69.7) | 0.512 |
| Lymphocyte (%) | 21.1 (18.0 – 35.0) | 28.0 (21.0 – 35.1) | 0.231 |
| Monocyte (%) | 7.0 (4.0 – 8.6) | 6.0 (5.0 – 8.0) | 0.730 |
| Eosinophil (%) | 0.1 (0 – 1.0) | 0.9 (0 – 1.7) | 0.075 |
| Basophil (%) | 0 (0 – 0.2) | 0 (0 – 0.4) | 0.233 |

The reference ranges for white blood cell, neutrophil, lymphocyte, and monocyte counts were 5-10 x 103 cells/µl, 45.0-74.0%, 16.0-45.0% and 0-10.0%, respectively.

**Supplementary Table S11** Correlation between white blood cell (WBC) counts and neutralizing antibody levels in COVID-19 patients

| Variants | Vaccination | Spearman’s correlation (r) and *p*-value of nAb levels against SARS-CoV-2 variants in COVID-19 patients | | | | | | | |
| --- | --- | --- | --- | --- | --- | --- | --- | --- | --- |
|  |  | Day 0 | | Day 14 | | Day 28 | | Day 365 | |
|  |  | r | *p*-value | r | *p*-value | r | *p*-value | r | *p*-value |
| Ancestral | Unvaccinated | -0.158 | 0.601 | 0.738 | 0.333 | 0.056 | 0.929 | 0.295 | 0.420 |
|  | Breakthrough | -0.060 | 0.663 | -0.157 | 0.398 | -0.341 | 0.029 | 0.093 | 0.612 |
| Alpha | Unvaccinated | -0.233 | 0.440 | 0.737 | 0.333 | 0.224 | 0.638 | 0.295 | 0.420 |
|  | Breakthrough | -0.010 | 0.942 | -0.159 | 0.394 | -0.348 | 0.026 | 0.074 | 0.686 |
| Beta | Unvaccinated | -0.219 | 0.468 | 0.738 | 0.333 | -0.585 | 0.167 | 0.419 | 0.239 |
|  | Breakthrough | -0.061 | 0.655 | -0.227 | 0.220 | -0.356 | 0.022 | 0.077 | 0.673 |
| Gamma | Unvaccinated | 0.026 | 0.932 | 0.738 | 0.333 | -0.580 | 0.176 | 0.419 | 0.239 |
|  | Breakthrough | -0.081 | 0.553 | -0.236 | 0.200 | -0.366 | 0.019 | 0.078 | 0.669 |
| Delta | Unvaccinated | -0.037 | 0.906 | 0.738 | 0.333 | 0.150 | 0.762 | 0.369 | 0.304 |
|  | Breakthrough | -0.083 | 0.544 | -0.159 | 0.393 | -0.355 | 0.023 | 0.065 | 0.723 |
| Lambda | Unvaccinated | -0.105 | 0.728 | 0.737 | 0.333 | -0.356 | 0.443 | 0.420 | 0.230 |
|  | Breakthrough | -0.111 | 0.418 | -0.143 | 0.445 | -0.344 | 0.027 | 0.074 | 0.688 |
| Mu | Unvaccinated | -0.153 | 0.613 | 0.730 | 0.321 | -0.412 | 0.367 | 0.394 | 0.272 |
|  | Breakthrough | -0.099 | 0.470 | -0.204 | 0.272 | -0.372 | 0.017 | 0.095 | 0.606 |
| Delta plus | Unvaccinated | -0.036 | 0.907 | 0.730 | 0.330 | 0.056 | 0.929 | 0.419 | 0.239 |
|  | Breakthrough | -0.073 | 0.595 | -0.192 | 0.302 | -0.366 | 0.019 | 0.039 | 0.833 |
| BA.1 | Unvaccinated | -0.003 | 0.997 | 0.711 | 0.298 | -0.430 | 0.348 | 0.443 | 0.211 |
|  | Breakthrough | -0.126 | 0.361 | -0.290 | 0.114 | -0.381 | 0.014 | 0.005 | 0.979 |
| BA.2 | Unvaccinated | -0.132 | 0.652 | 0.737 | 0.332 | -0.356 | 0.443 | 0.443 | 0.211 |
|  | Breakthrough | -0.204 | 0.136 | -0.303 | 0.098 | -0.415 | 0.007 | -0.016 | 0.931 |
| BA.5 | Unvaccinated | -0.132 | 0.652 | 0.949 | 0.167 | 0.037 | 0.943 | 0.492 | 0.160 |
|  | Breakthrough | -0.067 | 0.629 | -0.229 | 0.216 | -0.285 | 0.071 | 0.054 | 0.769 |
| XBB | Unvaccinated | 0.065 | 0.833 | 0.743 | 0.318 | -0.243 | 0.600 | 0.320 | 0.378 |
|  | Breakthrough | -0.193 | 0.157 | -0.261 | 0.156 | -0.307 | 0.051 | 0.062 | 0.735 |
| XBB.1.5 | Unvaccinated | -0.131 | 0.651 | 0.734 | 0.328 | -0.168 | 0.738 | 0.542 | 0.117 |
|  | Breakthrough | -0.207 | 0.130 | -0.209 | 0.260 | -0.384 | 0.013 | 0.045 | 0.809 |
